# Transcriptomic analysis of iPSC-derived endothelium reveals adaptations to high altitude hypoxia in energy metabolism and inflammation

**DOI:** 10.1371/journal.pgen.1011570

**Published:** 2025-02-10

**Authors:** Olivia A. Gray, David B. Witonsky, Jordan Jousma, Débora R. Sobreira, Alexander Van Alstyne, Ru-Ting Huang, Yun Fang, Anna Di Rienzo

**Affiliations:** 1 Department of Human Genetics, University of Chicago Division of the Biological Sciences, Chicago, Illinois, United States of America; 2 Department of Medicine, Section of Pulmonary and Intensive Care, University of Chicago Hospital: The University of Chicago Medicine, Chicago, Illinois, United States of America; University of California Santa Barbara, UNITED STATES OF AMERICA

## Abstract

Tibetan adaptation to high-altitude hypoxia remains a classic example of Darwinian selection in humans. Amongst Tibetan populations, alleles in the *EPAS1* gene *-* whose protein product, HIF-2α, is a central regulator of the hypoxia response - have repeatedly been shown to carry some of the strongest signals of positive selection in humans. However, selective sweep signals alone may only account for some of the phenotypes that differentiate high-altitude adapted populations from closely related lowlanders. Therefore, there is a pressing need to functionally probe adaptive alleles and their impact at both the locus-specific and genome-wide levels and across cell types to uncover the full range of beneficial traits. To this end, we established a library of induced pluripotent stem cells (iPSCs) derived from Tibetan and Han Chinese individuals, a robust model system allowing precise exploration of allelic effects on transcriptional responses, and we differentiated them into vascular endothelium. Using this system, we focus first on a hypoxia-dependent enhancer (ENH5) that contributes to the regulation of *EPAS1* to investigate its locus-specific effects in endothelium. Then, to cast a wider net, we harness the same experimental system to compare the transcriptome of Tibetan and Han Chinese cells in hypoxia and find evidence that angiogenesis, energy metabolism and immune pathways differ between these two populations with different histories of long-term residence at high altitude. Coupled with evidence of polygenic adaptations targeting the same pathways, these results suggests that the observed transcriptional differences between the two populations were shaped by natural selection.

## Author summary

Understanding the ways in which humans have adapted to extreme environmental conditions is of great interest to evolutionary biologists. For instance, substantial research has focused on characterizing how Tibetan populations have genetically adapted to conditions of low oxygen (hypoxia) at high elevation. Nevertheless, the inherent challenges of measuring unique traits or sampling relevant cell and tissues in such remote environments, limits our ability to assess Tibetan adaptation. Here we present the development of a model system in which to interrogate molecular differences between Tibetans and Han Chinese individuals – a closely related lowland population – in a cell type of interest. Specifically, we build a panel of Tibetan and Han Chinese induced pluripotent stem cells (iPSCs), from which we differentiate endothelial cells, a significant hypoxia-responsive cell type. We use these cells to test how Tibetan genetic ancestry influences the cellular response to hypoxia and find substantial differences between populations in metabolic and inflammatory responses. These findings not only help to inform our understanding of high-altitude adaption but can provide critical information for designing hypothesis-driven field studies in the future.

## Introduction

Tibetan populations provide a well-characterized example of human adaptations to local environments having lived in the highly hypoxic Tibetan plateau for over 10,000 years [1]. Adaptation to this environment has resulted in a bevy of phenotypes distinguishing Tibetan populations from closely-related acclimatized lowlanders, such as the Han Chinese, and/or from other long-term high altitude residents, such as Andeans [2–5]. Most notably, compared to East Asian lowlanders at high altitude, Tibetans exhibit lower rates of pregnancy complications (e.g., preeclampsia) and higher birth weights, a blunted erythropoietic response to hypoxia, no or modest pulmonary hypoxic vasoconstriction, and low rates of hypoxic pulmonary hypertension (PH) [5–8]. They also have lower rates of chronic mountain sickness (CMS) compared to Andean highlanders [4,5,9].

A few of these traits have been successfully mapped to specific genes [6,8,10], and the Tibetan genome has been well-characterized through numerous selection scans [3,6,10,11]. Recent functional investigations have shed light on the regulation of the *EPAS1* gene, which harbors one of the most robust signals of selection in the Tibetan genome. These studies revealed that alleles on the selected haplotype are linked to no or modest elevation of hemoglobin concentration [6,7,10,12], and disrupt multiple enhancers of *EPAS1* dampening the response to hypoxia across multiple cell and tissue types [13–15]. However, functional studies of adaptive genetic variation remain relatively sparse, and have been limited by logistical challenges in their ability to characterize the consequences of adaptive alleles in tissues of interest and in hypoxic conditions.

Beyond exploring the functional impacts of well-characterized candidate adaptive alleles, much remains to be understood about the evolved phenotypes, at the molecular and organismal levels, that allow Tibetans to survive in high-altitude hypoxia. Such evolved phenotypes can be identified by measuring traits at high altitude in cohorts of Tibetans and of a closely related low-altitude population while controlling for technical and other environmental confounders. Fieldwork studies have used this approach to identify a number of interesting candidates [4,9,16–18], but such searches for adaptive traits are limited to phenotypes that can be measured in the field and are often constrained by prior hypotheses. A complementary approach to fieldwork could search for evolved molecular phenotypes, e.g., differences in transcript levels across high and low altitude populations, measured *in vitro* in hypoxic conditions. However, the lack of cell culture models for the populations and the tissues of interested has, until recently, made this approach unfeasible.

Recent innovations now enable reliable and highly successful reprogramming of terminally differentiated cells into an induced pluripotent state. While historically used to study human disease [19–21], population panels of induced pluripotent stem cells (iPSCs) offer an unprecedented opportunity to study the functional and molecular impact of human adaptive alleles in specific environmental conditions and in a cell-type specific manner. This tool is ideally positioned to investigate cell types which are not easily accessible *in situ*. For instance, the vascular endothelium, which acts as a primary mediator of both the physiological and pathological responses to hypoxia [22,23] and has been directly implicated in Tibetan adaptation [13–15], is an important candidate for adaptations to hypoxia. While previous studies have examined Tibetan gene expression in the endothelium, these studies have been limited to accessible endothelial cell types, such as umbilical vein endothelial cells (HUVECs) which may not be an optimal model as they represent a highly-oxygenated, highly-permeable venous environment specific to fetal development [24,25]. To address this gap, we develop of an iPSC panel consisting of Tibetan and Han Chinese cell lines which we leverage to investigate hypoxic gene regulation in the vascular endothelium.

## Results and discussion

Here, we describe the development of a new resource, i.e., a panel of iPSC lines, and examples of its application to study Tibetan adaptations to hypoxia in the endothelium by: 1) editing a known regulatory element of the *EPAS1* gene [15], 2) testing for transcriptional differences between Tibetan and Han Chinese lines in normoxia and hypoxia, and 3) testing for polygenic adaptations to aid in the interpretation of between-population transcriptional differences.

### iPSC panel development & differentiation

We developed a panel of sex-matched Tibetan and Han Chinese iPSCs in order to characterize Tibetan molecular phenotypes as they compare to a closely related lowlander population. We used lymphoblastoid cell lines (LCLs) to generate iPSCs [26] as this cell type is publicly available for many 1,000 Genomes Project (1KGP) populations [27], offering the opportunity to assess the outcomes of positive selection in population samples that are well characterized at the genetic level. Among the 1KGP population panel, we chose 10 Han Chinese (CHB) LCLs as our comparison group because this population is genetically close to Tibetans, but do not have a history of long-standing residence at high-altitude. For the Tibetan cohort, we generated LCLs from the peripheral blood samples of 10 unrelated individuals of Tibetan origin residing in the Chicagoland area, henceforth referred to as the Tibetan Alliance of Chicago (TAC) samples. Genome-wide Single Nucleotide Polymorphism (SNP) genotype data were collected for all TAC samples, and their genetic ancestry was evaluated relative to that of previously published Tibetan individuals sampled in Tibet [6,28] (See Methods). We find that, despite being recruited in Chicago, Tibetans in our cohort showed strong genetic similarity to other Tibetans, in particular with those from the Western Plateau (S1 Fig). The 20 LCLs were reprogrammed into iPSCs in batches, balanced by population and sex, using the methods described by Burrows et al [29]. Following extensive QC (see Methods, S2 Fig and S1 Table) – including assessing inter-population differential RNA expression (S2 Table) – we successfully differentiated all 20 lines into vascular endothelium [30] (see Methods, S3, S4A and S5 Figs).

### CRISPR-modified iPSC-EC lines capture the impacts of an *EPAS1* enhancer in vascular endothelial cells

One advantage of iPSCs lies in their flexibility for targeted genetic modifications, enabling subsequent assessment of locus-specific effects in a cell type of interest. The possibility of introducing allelic modifications into a relevant genetic background is particularly important when trying to identify the functional consequences of candidate adaptive variation in the relevant cell types. We took advantage of this property to ascertain the effect of the previously identified *EPAS1* enhancer ENH5 within a Han Chinese genetic background, which is likely to be close to that of the ancestral population prior to adaptation to high altitude.

ENH5 had been previously identified by scanning the *EPAS1* region spanning the selection signals by means of reporter gene enhancer assays in endothelial cells cultured in normoxia and hypoxia, with each enhancer construct containing alternatively the low- or the high-altitude alleles. ENH5 was shown to have higher enhancer activity in hypoxia than normoxia and the high-altitude alleles conferred lower activity compared to the low altitude ones. ENH5 was shown to interact with the *EPAS1* promoter through promoter capture HiC and its deletion in immortalized aortic endothelial cells of European ancestry and in a mouse model resulted in the down-regulation of a large number of genes including known HIF-2α target genes [15].

Here, we replicated this deletion (chr2: 46578867 to 46579857; hg19) spanning the ENH5 enhancer in an iPSC line derived from individual CHB633, who carries the homozygous ancestral haplotype across *EPAS1*. Therefore, knockout cells in this system carry a low-altitude haplotype across the entire *EPAS1* locus, but with complete loss of ENH5 activity. These ΔENH5 iPSCs (subsequently referred to as knockout, KO) and the original CHB633 (wild type, WT) iPSCs were differentiated into vascular endothelium in five independent replicates (see Methods) and cultured in hypoxic conditions (1% O_2_) for 48 hours.

**Fig 1:**
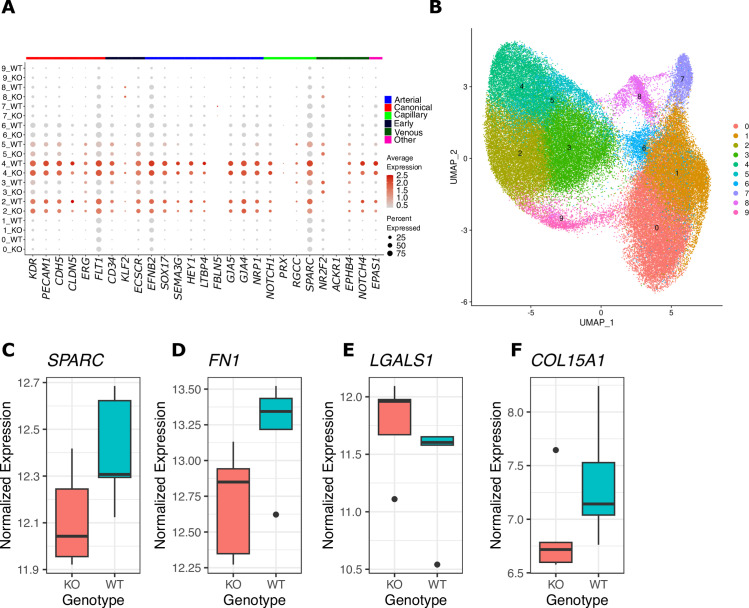
Single-cell RNA-sequencing of CRISPR-modified iPSC-ECs. A) Expression of endothelial marker genes across the 10 cell clusters (labeled 0–9) identified in the single cell data shown in B. Each row of the dot plot indicates the cluster number and the ENH5 genotype, the size and color of the dots indicate, respectively, the percent of cells expressing that gene and the average expression. Across both genotypes, clusters 2 and 4 robustly express endothelial marker genes. B) UMAP plot of iPSC-ECs colored by cluster. C–F) A selection of genes found to be significantly differentially expressed between KO and WT in the endothelial cluster (clusters 2 & 4). The data represents the pseudobulk counts in clusters 2 & 4 across 5 replicates per genotype which have been VST normalized.

To assess the impact of the ENH5 deletion in vascular endothelial cells, we performed single cell RNA-sequencing in a targeted 10,000 cells per replicate. We assessed the expression of 25 endothelial markers [31,32] (in the 10 UMAP clusters identified by Seurat [33–35] (Fig 1A and 1B). This analysis identified two cell clusters (clusters 2 and 4 in Fig 1B) as being robustly enriched in all endothelial markers; transcript data from the cells in these two clusters were pooled to compare expression differences between KO and WT in a cell-type specific manner (S3 Table indicates the number of cells per replicate in this pool). We also pooled clusters 2–5, all of which expressed canonical endothelial marker genes to some degree (Figs 1A, 1B, S4B and S4C), and found that the results of the two pooling schemes were highly correlated (S4D and S4E Fig). Differential expression analysis detected 49 DE genes across genotypes (adjusted *p* < 0.05, S6A Fig and S4 Table). While *EPAS1* downregulation did not meet transcriptome-wide significance (*p adj.* = 0.25), it was significant at the single-gene level [log fold change (logFC) = −0.32, *p.* = 0.0032, S6A Fig and S4 Table]. Moreover, previous research on the dynamic expression of *EPAS1* under hypoxic exposure suggests that downstream expression of HIF-2α targets is a more consistent readout of HIF-2α activity [15,22].

To more fully assess the effects of this deletion on HIF-2α targets, we compared the expression differences across between genotypes with those in primary human endothelium in which either HIF-1α or HIF-2α were stabilized and overexpressed [36]. Because ENH5 is an *EPAS1* enhancer, we expect HIF-2α rather than HIF-1α target genes to be predominantly DE in the comparison between WT and KO iPSC-EC lines cultured in hypoxia. As shown in S6B Fig, we found a significant negative correlation (Spearman’s r = −0.22, *p* = 2.29e-14) between the logFC of gene expression between KO and WT cells and that of genes that were preferentially HIF-2α regulated. Consistent with our expectation, no such correlation was observed with the logFC of HIF-1α regulated genes (*p* = 0.486). Amongst the most significantly DE genes between KO and WT, we identified well known HIF-2α target genes such as *CAV1* and *SLC2A1*, both of which have been shown to be upregulated in pulmonary hypertension [37–39]. Both genes are downregulated in the KO iPSC-ECs relative to WT lines suggesting that that loss of ENH5 activity protects against PH [15], which in turn is consistent with the lower rates of PH reported in Tibetan populations [37,38,40,41].

We next compared the results from our iPSC-ECs with previously published bulk RNA-seq differential expression data from an orthologous ENH5 KO mouse model [15]. Our goal was to identify pathways that are enriched for DE genes between KO and WT in hypoxic conditions *in vitro* and *in vivo*. We find that the greatest overlap of DE genes between human iPSC-ECs and mouse tissues (*lsfr* < 0.1 or adjusted *p* < 0.1) is with the mouse lung (overlapping DE genes = 13) and left atrium (overlapping DE genes = 8), consistent with the relatively high number of endothelial cells in these tissues and their importance in oxygen sensing. We performed gene set enrichment analysis on DE genes in iPSC-ECs, mouse lung, and mouse left atrium separately and find that the most significantly enriched gene set in the iPSC-EC data, Collagen-Containing Extracellular Matrix (ECM, GO:0062023, adjusted *p =* 9.38E-04), is also significantly enriched in both mouse tissues (adjusted *p =* 3.76E-04 lung, 1.62E-05 left atrium). Epithelial to Mesenchymal Transition (EMT, MsigDB Hallmark) is also significantly enriched across the three cell- and tissue-types (adjusted *p* = 0.0272 iPSC-ECs, 0.00461 mouse lung, 5.24E-13 mouse left atrium) (S7 Fig).

The shared enrichment signals of ECM and EMT are of particular interest, as they are both linked with pathogenic and normal developmental response to hypoxia in heart and lung endothelial cells [42–44]. Genes in these pathways, which are DE in the iPSC-ECs implicate ENH5 in protection against hypoxia-induced PH. These include: *SPARC* (Fig 1C) -- which is known to be upregulated in lungs during development of hypoxia-induced PH in mice [45] -- and is down-regulated in the KO relative to WT iPSC-ECs (Fig 1C), as well as *FN1* (Fig 1D), a central regulator of ECM organization, thought to be involved in the pathogenesis of PH [46–48]. Additional genes, particularly in the ECM pathway, implicate ENH5 in angiogenesis. For example, *LGALS1* (galectin1) is a hypoxia-induced pro-angiogenic factor that is upregulated in KO relative to WT (Fig 1E), suggesting that lower activity of ENH5 provides greater angiogenic potential. Consistent with this proposal, *COL15A1*, which codes for a precursor of the antiangiogenic protein Restin [49] is down-regulated in KO relative to WT (Fig 1F). The consistency between an *in vitro* system of vascular endothelial cells and an *in vivo* model with orthologous deletions of the same enhancer underlines the tractability of iPSCs in capturing cell-type specific expression differences that may be reflective of *in vivo* physiological effects.

### Transcriptome-wide analysis of iPSC-ECs captures between-population differences linked to energy metabolism and inflammation

We next expanded our scope beyond locus-specific effects to identify molecular phenotypes that evolved in response to hypoxia as populations adapted to the high-altitude environment. These molecular traits may offer insights into novel beneficial organismal traits to be tested in future field studies, thus facilitating a more comprehensive understanding of the evolutionary physiology of Tibetan populations.

To this end, we utilized our complete panel of 20 cell lines to compare transcript levels between TAC and CHB iPSC-derived vascular endothelium under both normoxic and hypoxic conditions. Given the larger number of cell lines and treatment conditions, we performed bulk RNA-sequencing rather than single cell RNA-sequencing. We further purified iPSC-ECs through a pulldown protocol using beads coated with an antibody for a canonical surface cell marker of the vascular endothelium (CD144/*CDH5*), as outlined in [30] and described in the Methods section. This experimental design allowed us to culture the purified vascular endothelial cells in parallel in normoxia (20% O_2_) and hypoxia (1% O_2_) for 48 hours prior to harvest and transcriptional profiling. We therefore generated two transcriptomes (one under normoxia and one under hypoxia) per individual, and leveraged the DREAM (differential expression for repeated measures) method, which enhances power while controlling false positives in multi-measure experimental designs [50]. In addition to known covariates, sources of unknown variation were modeled using surrogate variable analysis [51]. Differential gene expression was assessed between treatment conditions (Hypoxia – Normoxia), between populations (TAC – CHB), and the interaction of the two (response).

Our single cell RNAseq data described above showed that *CHD5* is expressed, albeit to a lesser extent, in clusters beyond the defined endothelial clusters 2 and 4 (S4B and S4C Fig). Therefore, we decided to characterize the cell type composition of the bulk RNAseq data and its variability across lines and across populations using transcriptomic signatures from previously identified clusters in our scRNA-seq data. A systematic difference in cell type proportions across TAC and CHB lines could confound the analysis of differential expression across populations. To that end, we deconvoluted the bulk RNA-seq data for each line using the transcriptional information for the clusters identified in the single cell experiments (See Methods, [52]. This analysis revealed that across all lines a mean of 72% of all cells were inferred to belong to the endothelial clusters 2 and 4 (S5 Table). The proportion of inferred endothelial cells were highly consistent across conditions for each line (S8A Fig). While there was variability in cell type proportions amongst lines, these were not found to be correlated with PluriTest pluripotency scores (S8B and S8C Fig); nor were there significant differences in cell type proportions between populations (*p* = 0.37, S8D Fig). Additionally, we found that the top surrogate variable included in our differential expression model is significantly correlated with the proportion of cells in the endothelial clusters 2 and 4 (S8E Fig, r = 0.77, *p* = 6.158e-9) thus correcting for any remaining confounding effects of cell type composition.

**Fig 2:**
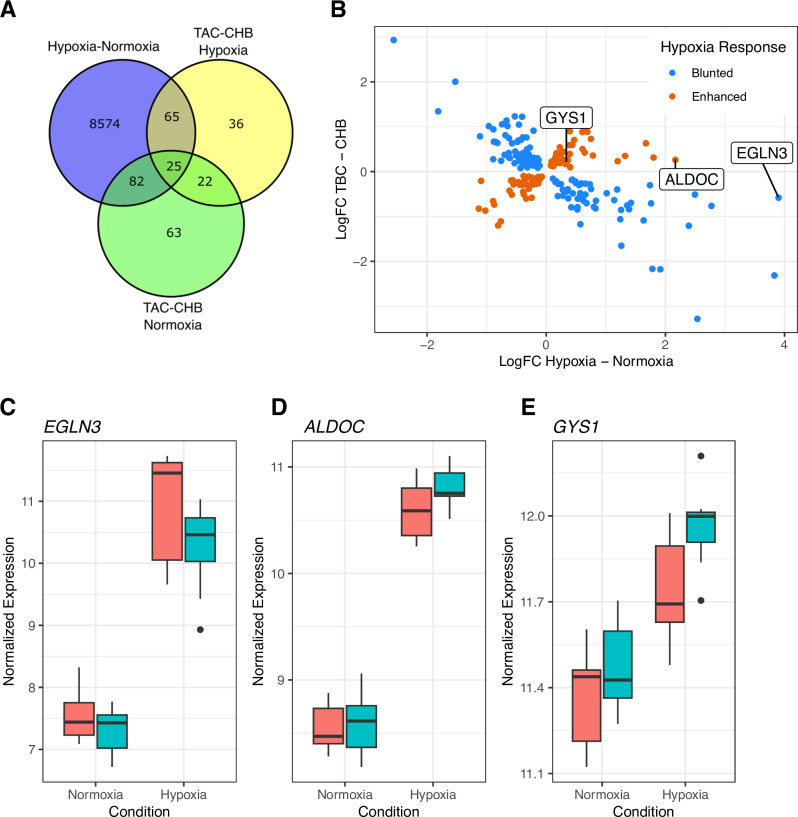
Between-population comparison of transcriptional response to hypoxia in iPSC-ECs. A) Venn diagram showing the overlap of DE genes in each of the three comparisons described. For gene set enrichment analysis, we focused on all genes DE between populations in hypoxia (148 genes; yellow circle), the set of genes DE between populations in both normoxia and hypoxia (47 genes; overlap between yellow and green circles), and DE genes between populations that respond to hypoxia (overlap of yellow and purple circles). B) Transcriptome-wide comparison of response to hypoxia between TAC and CHB iPSC-ECs. The horizontal axis shows the difference in transcript levels (logFC) between hypoxia and normoxia for each gene whereas the vertical axis shows the difference in transcript levels (logFC) between populations in hypoxia. Blue points indicate genes showing a blunted response to hypoxia in TAC relative to CHB iPSC-ECs; conversely, orange points indicate genes with an enhanced response to hypoxia in TAC vs. CHB iPSC-ECs. The 3 genes shown in C–E are indicated. C) *EGLN3*, a direct target of HIF-2α in endothelium, displays a blunted hypoxia response in TAC relative to CHB iPSC-ECs. While it is up-regulated in hypoxia in both populations, its expression is signficantly lower in TAC iPSC-ECs in hypoxia than CHB (adjusted *p* = 0.007). D and E) Both *GYS1* and *ALDOC* display an enhanced hypoxia response in TAC iPSC-ECs. While both are up-regulated in hypoxia, they are found to be expressed at higher levels in TAC iPSC-ECs relative to CHB in this condition (*P =* 0.005 and *P =* 0.07 respectively).

As expected, our analysis identified the largest number of DE genes between conditions (Hypoxia – Normoxia; n = 8,746 out of 18,311 expressed genes, adjusted *p* < 0.05). No significant differences in the response to hypoxia were observed between the two populations, likely due to limited statistical power. To identify the biological functions that differ between populations, we performed gene set enrichment analysis in the following groups of DE genes: genes that are DE between populations in hypoxia (n = 148), genes that are DE in both oxygen concentrations (n = 47), and hypoxia-responsive genes which are DE between populations in hypoxia (n = 90) (Fig 2A and S6 Table). Each set of DE genes allowed us to probe specific aspects of evolved transcriptional regulation in Tibetan populations.

We first examined the group of DE genes between populations in hypoxia as these are most likely to be relevant to adaptations to a hypoxic environment. Moreover, considering the rapid post-translational degradation of the HIFs in normoxia, only differences observed in hypoxia are likely to be linked to the canonical transcriptional response to hypoxia. Consistent with our previous findings, both the *EPAS1* gene and the HIF-2α target gene *EGLN3*, were significantly DE between populations in hypoxia (adjusted *p* = 0.001 and 0.007, respectively) but not significantly different in normoxia (adjusted *p* = 0.193 and 0.450 respectively; Figs S9A, 2B and 2C). This group of DE genes are enriched in the Glycolysis gene set (MsigDB Hallmark, adjusted *p* = 7.57E-04, S10 Fig and S7 Table), which aligns with the profound impact that physiological hypoxia is known to have on energy production, particularly in the endothelium, where cells heavily rely on glycolysis [53].

It has been proposed that natural selection in Tibetans favored a dampening of the acclimatization response to hypoxia, a process called “maladaptive plasticity” [54]. The unelevated Hb levels in Tibetans are considered a prime example of dampened response, which results from the adaptive alleles at *EPAS1*. However, the response to hypoxia involves multiple physiological systems, from respiratory to cardiovascular to muscular, that function in concert to compensate for the drop in oxygen pressure at high altitude. Therefore, it can be hypothesized that while natural selection favored a dampening of the response in some physiological compartments, it may concomitantly have favored an enhanced response in others to compensate. To test this idea, we focused on the 90 genes that were DE in hypoxia between populations as well as DE in response to hypoxia in iPSC-ECs (adjusted *p* < 0.05) and asked whether Tibetans exhibit a blunted or an enhanced response to hypoxia relative to that of Han Chinese. In a blunted response, genes that are upregulated in hypoxia (positive logFC between treatment conditions) tend to increase in expression less in Tibetans than in Han Chinese whereas genes downregulated in hypoxia have higher transcript levels in Tibetans compared to Han Chinese in hypoxia, resulting in a negative logFC between populations (Fig 2B and S6 Table). In an enhanced response, genes that are upregulated in hypoxia have higher expression levels in Tibetans compared to Han Chinese and conversely genes that are downregulated in hypoxia have lower expression in Tibetans. To investigate the nature of the differences between TAC and CHB, we plotted the logFC in expression between populations (TAC-CHB) against the logFC in expression between conditions (Hypoxia – Normoxia), expanding our scope to genes with adjusted *p* < 0.1 in both conditions (Fig 2B). At this significance cutoff, we found that 57% of DE genes between populations in hypoxia (128 out of 224) are negatively correlated in effect size, consistent with a blunted response to hypoxia in Tibetans (Fig 2B). Whether the blunted response observed in Tibetan iPSC-ECs reflects only the downregulation of the HIF-2α cascade due to regulatory variants in *EPAS1* or the result of additional genetic variation across other genes is unclear.

Interestingly, 96 out of 224 genes (42.9%) have the opposite trend, i.e., they have an enhanced response to hypoxia in Tibetan endothelium compared to Han Chinese. Looking more closely, we found that these genes were also enriched in Glycolysis-related gene sets (MSigDB Hallmark, adjusted *p* = 0.005), and included several key genes that, when upregulated, are predicted to enhance glycolysis and glycolytic energy stores in the endothelium, such as the primary glycogen synthase gene (*GYS1*) [55,56]. Another example, *ALDOC* codes for an essential enzyme in the glycolysis pathway [57], which is upregulated in a hypoxic cellular environment [58]. This pattern of an enhanced response to hypoxia in Tibetans is novel. Perhaps because of the focus on variation at the *EPAS1* gene, much of the research to date has described a dampened response to hypoxic exposure. Our results, however, raise the possibility that adaptation in Tibetans involved also an enhancement of some of the physiological processes that change in response to high altitude exposure to maintain homeostasis in the face of hypoxic stress. The nature of such enhanced processes needs to be investigated in larger *in vitro* and *in vivo* studies.

Finally, to better understand transcriptional effects extending beyond the HIF regulatory axis, we examined the group of 47 DE genes between populations in both normoxia and hypoxia (adjusted *p* < 0.05 in both conditions, Fig 2A and S6 Table), predicting that genes in this group are less likely to be driven by the HIF pathway but may still have an impact on Tibetan physiology. Overall, we observed a strong correlation in log-fold change (logFC) between populations (TAC-CHB) under both conditions, indicating that amongst these genes the differences between populations are robust to differences in oxygen concentration (S9B Fig). In contrast to the genes that differ between populations only in hypoxia, the DE genes in both conditions were enriched for gene sets relating to inflammatory processes including Inflammatory Response (MSigDB Hallmark, adjusted *p* = 0.0176, S7 Table), Cellular Response To Molecule Of Bacterial Origin (GO:0071219, adjusted *p* = 0.0309), and Response To Lipopolysaccharide (GO:0032496, adjusted *p* = 0.0473, S7 Table). For example, *AXL* and *TLR2*, two canonical immunoregulatory genes, belong to this group and Tibetan iPSC-ECs exhibited higher expression of both genes than Han Chinese iPSC-ECs (S9C and S9D Fig). Both *AXL* and *TLR2* are cell surface proteins associated with inflammatory processes such as endothelial permeability, innate immune function, and trans-endothelial migration of immune cells. Both have also been linked to angiogenic activity such as promoting microvascular angiogenesis, and enhancing tube formation [59–63]. Therefore, these findings raise the possibility that Tibetans have an enhanced angiogenic capacity and inflammatory responses.

### Polygenic adaptations also point to beneficial traits in energy metabolism and inflammation

Testing for transcriptome-wide differences between TAC and CHB iPSC-ECs allowed us to point to adaptive molecular phenotypes that are probably due to genetic changes at many genes across the genome. As a complementary genome-wide analysis, we searched the Tibetan genome for evidence of beneficial traits resulting from polygenic adaptations. To this end, we used the GWAS results obtained by the BioBank Japan (BBJ) consortium [64] and allele frequency data for 344 Tibetans and 101 Sherpa (a Nepali population of Tibetan ancestry well-known for their mountaineering skills) as described in Jeong *et al* 2018 [6].

**Fig 3:**
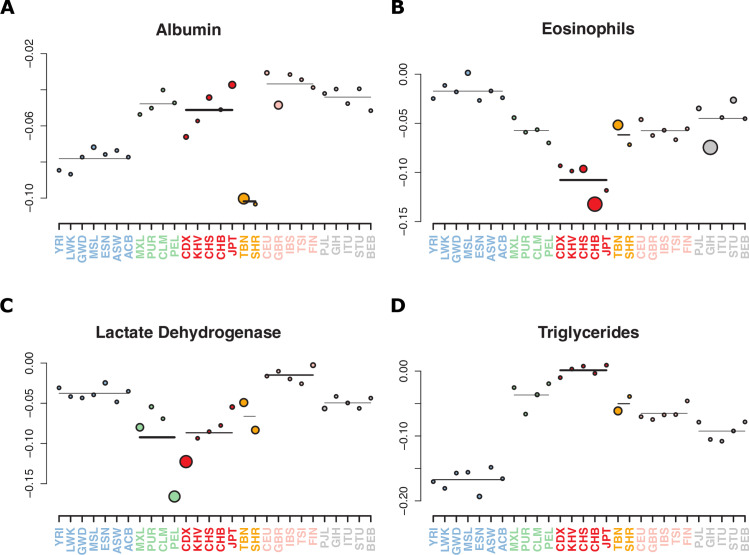
Signals of polygenic adaptation in Tibetan and Sherpa populations. The results of the multi-population test for polygenic adaptation from Berg & Coop, 2014 [66] are shown. These plots display the genetically predicted value of the trait indicated in the title using GWAS SNPs at a significance threshold of *p* < 1e-06. Each color corresponds to a geographic region containing multiple population cohorts. The regional average of the predicted values is plotted as a solid bar, with darkness and thickness proportional to the regional *Z* score. The genetically predicted value for each population is plotted as a colored circle, with circle size proportional to the population specific *Z* score. For all four traits, Tibetan and Sherpa populations (yellow) are significant outliers (*p* < 0.05) relative to other *p*opulation groups *P*-values for all tests and traits are shown in S8 Table.

We used two methods designed to detect consistent changes in the frequency of alleles associated with a trait of interest. The first approach considers the frequency difference of the trait-increasing alleles in a pair of populations, specifically Tibetans and 1KGP CHB; the results are compared to 10,000 sets of control SNPs [65]. The second approach [66] calculates a genetic value for a trait of interest in each of a set of populations by summing up the product of the frequency at each Genome-Wide Association Study (GWAS) SNP and the effect size of that SNP and it compares GWAS-ascertained SNPs with a large number of control SNPs. Specifically, we used the regional “outlier” test to asks if the genetic value of a trait in Tibetans and Sherpa, as a regional group, is significantly different from that of the other populations. The results are shown in Figs 3 and S11 and S8 Table.

Among the significant signals of polygenic adaptation, we found evidence across tests for adaptive changes towards lower serum albumin and triglyceride levels (Fig 3A and 3B), and higher lactate dehydrogenase and eosinophil count (Fig 3C and 3D) in high-altitude adapted populations compared to low-altitude populations (Fig 3). Interestingly, the results for serum albumin and triglyceride levels are consistent with two recent reports of observed phenotypic differences between Tibetan and Han Chinese at high-altitude [8,67]; for both traits, the observed differences between populations were statistically significant and in the same direction as the differences predicted by the polygenic adaptation tests. Importantly, many of these signals corroborate the results of our transcriptional analyses pointing towards adaptive changes in both energy metabolism and inflammation pathways.

For instance, the signals for decreased triglyceride (TG) and increased lactate dehydrogenase (LDH) levels (Fig 3B and 3C) both point to a shift in the energy production axis. In hypoxic conditions, triglycerides are produced faster than they can be consumed, as cells shift from oxidative phosphorylation to glycolysis. Notably, endothelial cells rely heavily on glycolysis under all conditions, and this reliance increases under hypoxia [68]. In our analysis of expression differences between Tibetan and Han Chinese endothelium under hypoxia, we find that *STC1*, which is linked to increased triglyceride levels [69], is downregulated in Tibetans while expression of *GYS1*, which synthesizes glycogen, is increased (S6 Table and Fig 2D). In this context, the observed adaptation signal towards elevated LDH in Tibetans is consistent with an evolutionary advantage of decreasing triglyceride production while increasing glycogen storage and lactate production, thus optimizing energy metabolism in the challenging conditions of high-altitude hypoxia. Alternatively, the polygenic adaptation signal for LDH could be an evolutionary mechanism to compensate for the blunted HIF-2α response to hypoxia observed in Tibetans due to the selected haplotype at *EPAS1*. Intriguingly, TG levels were reported to be positively correlated with fundal height in pregnant Tibetan women, which might imply that lower TG levels predict poorer reproductive outcomes in this population [8]. Additional research efforts are necessary to fully elucidate the complexities of Tibetan metabolic homeostasis and its connection to reproductive success.

In addition to previously reported phenotypes, we find a novel polygenic adaptation signal for elevated eosinophil count in Tibetan populations. Eosinophil elevation is commonly concomitant with immune response and is facilitated by the crosstalk between endothelial cells and eosinophils. This cross-talk has been well-documented as contributing to functions ranging from inflammatory response to angiogenesis, and is mediated by a cell surface marker known as Gal-3, which is expressed both on the endothelial and the eosinophil cell surface (*1*, *2*). Notably, we find that Gal-3 (*LGALS3*) is the most significantly differentially expressed gene between TAC and CHB iPSC-ECs under hypoxic conditions - where it is upregulated in Tibetan relative to Han Chinese endothelium (logFC = 0.95, adjusted *p* = 4.1e-5, S6 Table) - and both immune response and angiogenesis are implicated in enrichment analyses (S7 Table).

Finally, while not directly related to our endothelial transcriptomic results, the polygenic adaptation signal for lower albumin levels is interesting in its own right. Albumin acts as a regulator of plasma volume, with low albumin levels leading to an increase in plasma volume [70,71]. Recent work has shown that plasma volume expansion may account for the lower hemoglobin concentrations observed in Sherpa populations [72]. Moreover, during pregnancy, hypoalbuminemia may increase the expansion of maternal plasma volume, which is especially essential in low oxygen conditions [70]. In a Mendelian randomization analysis, He et al. found that lower albumin levels are causally linked to larger fetal placental volume, which in turn is a predictor of better reproductive outcomes [8]. These tantalizing findings taken together raise the possibility that modulation of albumin levels through polygenic adaptations may influence reproductive success at high altitude.

In summary, iPSC-derived cells provide numerous advantages for identifying candidate adaptive traits at both the cellular and molecular levels. They allow for the investigation of multiple cell types within the same individuals and in environmental conditions that closely mimic the local selective pressures acting on a population. Moreover, owing to transcriptome-wide profiling, the search for adaptative traits can be conducted in a hypothesis-free manner. Certainly, the study of local adaptations requires going beyond cellular and molecular phenotypes to include organismal traits, among which reproductive success is of primary importance. Nonetheless, iPSC-based approaches allow the detection and dissection of context-specific adaptation at the level of the cell state, cell type, and composite tissue/organoids to shed light on medically relevant human fitness. When paired with polygenic adaptation analyses, as done here, they can convincingly nominate candidate adaptive organismal traits to be examined in subsequent fieldwork. For example, our results suggest that future fieldwork could focus on immune and metabolic profiling in Tibetan populations as compared to closely related, acclimatized lowlanders. At the molecular level this could take the form of profiling inflammation markers or assessing variation in immune cell type proportions in adapted and non-adapted high-altitude populations. Meanwhile, metabolic adaptations could be probed through glucose-tolerance testing or comprehensive metabolic panels in high-altitude individuals to ascertain population-based differences in lipid levels and glucose metabolism. Given the challenges of conducting population fieldwork, particularly in remote areas, the ability to restrict the number of candidate traits to a subset with prior evidence from an unbiased search is a significant advantage.

## Materials and methods

### Ethics statement

Tibetan samples were obtained with formal informed written consent from Tibetans living in the Chicagoland area at the Tibetan Alliance of Chicago (TAC). The human subjects protocol was approved by the Institutional Review Board of the University of Chicago (IRB16-1501).

### Study sample and participant details

All subjects were either born in Tibet or were the descendants of four Tibetan-born grandparents. Peripheral whole blood samples were collected by venipuncture, and LCLs were generated at the University of Chicago using a standard protocol (Coriell Institute) for establishment of lymphoblastoid cell cultures from peripheral blood mononuclear cells (PBMCs). All Han Chinese samples used in the study were obtained from Coriell in the form of LCLs. In total 10 LCL lines from each ancestry group (TAC and CHB) were selected for iPSC generation. The samples were sex-balanced with 5 male and 5 female participants from each population group. Due to variable timing of collection and DNA extraction, the samples were genotyped across multiple platforms. Eight of the samples (TAC86, TAC252, TAC462, TAC540, TAC750, TAC801, TAC841, and TAC988) were genotyped using the Infinium Multi-Ethnic Global-8 v1.0 chip (Illumina, WG-316-1001), TAC859 was genotyped on the Infinium Multi-Ethnic AMR/AFR-8 v1.0 chip (Illumina, 20001090), and TAC569 was genotyped on the Infinium OmniExpress-24 v1.3 chip (Illumina, 20024631). CHB samples were purchased from the Coriell Institute and were originally collected and genotyped by the HapMap Project, sequenced by the 1,000 Genomes project, and contributed with consent to the NHGRI Sample Repository for Human Genetic Research [27,73].

Reprogramming of LCLs to iPSCs was performed in accordance with Thomas et al., 2015 [26]. Briefly, LCLs were nucleofected with episomal plasmids encoding OCT3/4, shP53, Lin28, SOX2, L-MYC, KLF4, and GFP (Addgene plasmids 27077, 27078, 27080, 27082) using the Amaxa transfection program X-005. Following transfection, cells were grown in suspension for a week before being transferred onto gelatin-coated plates containing CF-1 irradiated mouse embryonic fibroblasts (Gibco, A34180). Throughout this process cells were grown in hESC media (DMEM/F12 supplemented with 20% KOSR, 0.1 mM NEAA, 2 mM GlutaMAX, 1% Pen/Strep, 0.1 mM BME, and 12.5 ng/mL human bFGF), which was additionally supplemented with 0.5 mM sodium butyrate on days 2–12. After a minimum of 10 passages on CF-1 Mouse Embryonic Fibroblasts (MEFs), iPSCs were converted to feeder-free maintenance conditions on Matrigel (0.1 mg/ml) coated plates in MteSR1 (Stemcell Technologies, 85850) or Essential 8 media (Gibco, A1517001). Lines were fed fresh media daily and routinely passaged using Cell Release, an EDTA-based solution described in Chen et al., 2011 [74], and re-plated in media supplemented with Rho-associated kinase (ROCK) inhibitor Y27632 (BioVision, 1994-1). Following 10 passages in feeder free conditions cells were frozen and tested for freeze-thaw recovery. All generation and maintenance of iPSCs were performed in atmospheric oxygen concentration. Cells were grown in culture at 37°C, 5% CO_2_ in a standard incubator.

### Development & QC of iPSCs

The 20 LCLs (10 TAC and 10 CHB) were reprogrammed into iPSCs in batches, balanced by population and sex, using the methods described by Burrows et al [29]. Standard quality control tests of pluripotency and stability were performed. Specifically, to assess expression of endogenous and exogenous pluripotency factor, cell pellets were collected and flash frozen from each line as LCLs at day 0 prior to nucleofection, at day 8 prior to transition to CF-1 plates, at passage 10 as feeder-dependent iPSCs on CF-1 plates, and at passage 10 as feeder-free iPSCs on Matrigel. RNA was extracted using Rneasy Plus Mini kit (Qiagen, 74134) in a Qiagen Qiacube. cDNA was generated using the iScript cDNA synthesis kit (biorad, 1708891) and RT-qPCR performed on a QuantStudio 6 Flex machine using qPCRBIO SyGreen Blue Mix Lo-ROX (PCR Biosystems, PB20.11-05). RT-qPCR was used to identify the production of endogenous pluripotency factors (*OCT3/4*, *SOX2*, *KLF4*, *L-MYC*, *NANOG*, and *Lin28*) and the absence of plasmid-derived transcripts (Addgene plasmids 27077, 27078, 27080, 27082) using primers from Thomas et al., 2015 [26]. Plasmid-derived transcripts can be uniquely identified from endogenous pluripotency factors based both upon their unique sequence and the *EBNA-1* gene encoded in the vector backbone. *EBNA-1* is also encoded by the EBV used to immortalize the LCLs. For plasmid-derived factors, expression was measured relative to a random day 8 line and for *EBNA-1* expression, which measures both plasmid backbone and EBV presence, expression was measured relative to a randomly selected LCL line. For endogenous pluripotency factors, expression was measured relative to either an LCL line or a day 8 line. All expression was normalized relative to *RPLP0* [75]. Most lines had fully ejected the plasmids by passage 10 on feeder free, those that still showed a slight signal from the plasmid factor RT-PCR were re-tested at passage 10 post-conversion. At this point, all lines had ejected all episomal plasmids. All 20 lines were found to express similar levels of endogenous pluripotency factors.

Embryoid body assays were carried out to assess functional pluripotency in accordance with Romera et al. [76]. Briefly, feeder-dependent iPSCs grown in bFGF-containing hESC media were detached and manually transferred to polyhema-coated plates where they were grown in suspension for 7 days in bFGF-free hESC media. They were then moved to gelatin-coated plates and allowed to attach and grow in EB media (DMEM + 10% FBS) for an additional 2 weeks. The embryoid bodies were then fixed in PBS containing 4% paraformaldehyde and immunofluorescently stained to detect endoderm (alpha-Fetoprotein, 1:100, SC-130302, Santa Cruz Biotech; and HNF3β, 1:50 sc-374376, Santa Cruz Biotech), mesoderm (α-smooth muscle actin, 1:1500, CBL171, Millipore), and ectoderm (nestin, 1:250, SC-71665, Santa Cruz Biotech) lineages.

G-banded karyotyping was carried out by WiCell (Madison, WI) using standard protocols [77]. Four of the 20 lines had clonal mosaicisms including chromosomal abnormalities that are recurrent in pluripotent stem cells (S1 Table). These lines TAC462, TAC252, CHB619, and CHB557 are evenly divided amongst Tibetans and Han Chinese. Inclusion or exclusion of these lines did not substantially alter downstream results for any of our bulk RNA-seq experiments (S12 Fig). The iPSC line used for editing the ENH5 enhancer had normal karyotype.

Inter-population differences in gene expression in the iPSC lines was assessed using bulk RNA-sequencing data. Such differences, if any, could affect differentiation potential and, hence, confound downstream analyses comparing molecular phenotypes in the two populations. We find remarkable consistency across populations in expression levels of all six pluripotency factors – *NANOG*, *LIN28*, *KLF4*, *MYC*, *SOX2*, and *OCT3/4* (*POU5F1*) (S2B Fig). In addition, we used a bioinformatic assay (PluriTest) to assess pluripotency by uploading FastQ files of the RNA-seq data obtained in the iPSCs to the https://www.pluritest.org web site and analyzed according to standard settings [78].

### CRISPR-CAS9 editing of iPSCs

Enhancer region ENH5 was deleted in iPSC line CHB633 using an IDT ALT-R Ribonucleoprotein delivery method (RNP, IDT). We used guides described in [15], which had previously been used successfully to delete ENH5 in endothelial cells. Complexed RNPs were delivered to iPSCs in suspension using the Amaxa 2b-Nucleofector device (Bioscience Lonza) with the program A23 and plated at low density in 10 cm dishes to allow for single cell colony formation as described in [79]. Colonies were screened for the presence of a deletion fragment using previously described PCR amplification [15].

### Differentiation of iPSC-derived vascular endothelial cells

iPSCs were differentiated into vascular endothelium according to the protocol described in [30] with small adjustments. Our panel of TAC-CHB iPSCs were grown to confluence in batches of 4–8 lines (balanced for population and sex). On day 0 cells were detached using Cell Release, an EDTA-based solution described in [74], counted, and replated at a density of ~50,000 cells/cm^2^ in Essential 8 media supplemented with Rho-associated kinase (ROCK) inhibitor Y27632 (BioVision, 1994-1). On day 1, medium was replaced with N2B27 medium – a 1:1 mixture of DMEM:F12 (Life technologies, 11320-033) and Neurobasal media (Life technologies, 21103049) with glutaMAX (Life technologies, 35050038), β-Mercaptoethanol (0.097%) (Life technologies, 21985023), N2(Life technologies, 17502048), and B27 minus vitamin A (Life technologies, 12587010)] this was supplemented with 8 uM CHIR (Tocris, 4953) and 25 ng/ml hBMP4 (PeproTech, 120-05ET) and added to the cells at high volume (i.e., 15 mL in 22.1 cm dish). The cells were left undisturbed for 3 days. On day 4, media was exchanged for EC Induction Medium - StemPro-34 SFM medium (Life technologies, 10639-011) supplemented with 200 ng/mL VEGF165 (PeproTech, 100-20) and 2 μM forskolin (Abcam, ab120058), and penicillin-streptomycin]. EC Induction Medium was refreshed on day 5, and on day 6 CD144+ cells were either isolated via pulldown or replated for subsequent single-cell sequencing.

### Bead-based purification and characterization of iPSC-derived vascular endothelial cells

ECs were dissociated using Accutase (STEMCELL Technologies, 7920) and pulldown performed with the quadroMACS Separator (Miltenyi Biotec, 130-091-051) using CD144 MicroBeads (Miltenyi Biotec, 130-097-857). Isolated cells were seeded at ~26,000 cells/cm^2^ on plates coated in 2 µg/cm^2^ fibronectin (Fisher, 356008) in Expansion Medium - StemPro-34 SFM medium (Life technologies, 10639-011) supplemented with 50 ng/mL VEGF165 (PeproTech, 100-20). Cells were allowed to expand for 4 days, with Expansion Media refreshed every 24 hrs, then switched in EGM-2 for further expansion and cryopreservation, while the remaining cells were placed into either hypoxia (1% O_2_, 5% CO_2_ – Coy Labs Hypoxia Glove Box) or normoxia (~20% O_2_, 5% CO_2_ –standard lab incubator) for 48 hours.

Two iPSC-derived endothelial lines, one Tibetan (TAC801) and one Chinese (CHB608) were selected to assess the presence of additional endothelial markers. Following pulldown and expansion, the lines were fixed in the wells of a 6-well cell culture plate with PBS containing 4% paraformaldehyde. They were stained with antibodies against CD31/PECAM-1 (Invitrogen) and VWF (Invitrogen). The samples were then counterstained with various Alexa fluorophores visually assessed on an EVOS FL Digital Inverted Microscope (Advanced Microscopy Group) for presence and localization of the two proteins. In both lines CD31 was visible on the cell surface of all cells visualized, while VWF was seen in the cytoplasm clustered into prototypical storage granules known as Weibel–Palade bodies [80] (S3 Fig).

### iPSC-derived EC harvest for bulk and single cell RNA-sequencing

For single cell sequencing, CRISPR-modified cells were subjected to differentiation, but were harvested prior to bead purification as described in [30] to avoid disturbing the microfluidic processes of single-cell sequencing. Instead on day 6 of differentiation, these cells were passaged to fibronectin coated plates and allowed to rest for 24 hours at which point they were moved to the hypoxia chamber (1% O_2_) for 48 hours. Cells were detached in hypoxic conditions after which they were kept on ice to prevent reperfusion response and processed according to 10x recommendations.

For the population-wide bulk RNA sequencing, bead-purified iPSC-derived endothelial cells were washed and lysed in-plate using RLT Plus buffer from the Qiagen Rneasy Plus Mini Kit (74134). Cell culture replicates in hypoxia were lysed and harvested in the hypoxia box with no exposure to normoxia prior to lysis. Lysates were flash frozen and stored at −80C degrees until all batches were complete. RNA extraction was performed in 5 balanced batched of 8 (balanced for population, sex, and treatment) in a Qiagen Qiacube (9001292) using the Rneasy Plus Mini Kit. RNA was assessed for concentration prior to library preparation. 500 ng of each sample was used for library prep using Illumina TruSeq2 RNA-seq library prep kit and set A indexes (RS-122-2001). All libraries were assessed via bioanalyzer to be of sufficient quality, concentration, and fragment size for further processing. Samples were run across 5 lanes of the Illumina HiSeq4000 at the University of Chicago Genomics Core, with 8 balanced samples per lane resulting in an average of 51 M single-end reads per sample (S9 Table).

### Data analysis

#### Tibetan genetic ancestry characterization.

Genome-wide Single Nucleotide Polymorphism (SNP) genotype data were collected for all TAC samples, and their genetic ancestry was evaluated relative to that of previously published Tibetan individuals sampled in Tibet [6,28]. Tibetan genotype data was imputed using IMPUTE2, keeping genotypes with posterior probability ≥ 0.9. Phased genotype calls from whole genome sequencing of 59 high altitude Tibetan and Sherpa individuals (reported in [6]) and phase 3 1,000 Genomes Project data were used as imputation reference. Genotype data from the 10 TAC samples was merged with the data from 80 samples from 4 1,000 Genome Project populations (20 GIH, 20 KHV, 20 JPT and 20 CHB), 20 ethnically Tibetan samples from two districts in Nepal (10 Mustang district and 10 Gorkha district) [6] and 97 modern samples from 10 Tibetan populations (5 Gannan, 12 Chamdo, 4 Xunhua, 20 Gangcha, 9 Lhasa, 8 Nagqu, 10 Yajiang, 10 Shigatse, 10 Xinlong, and 9 Shannan) [28]. Starting from an initial set of 315,877 SNPs found in the overlap between those on the Human Origins array and those genotyped in our sample, we used PLINK v2.0 SNPs to further filter for a MAF > 0.05 across all 207 samples. Both admixture and PCA analyses were performed on this set of 207 samples and the resulting 174,246 SNPs. The program admixture v1.3.0 was run in unsupervised mode with k values ranging from 1 to 10 [81]. Though the admixture cross-validation (CV) error had its lowest value for k = 3 (0.57365), a value of k = 4 had only a slightly higher CV value (0.57853) and seemed to better distinguish the 1,000 Genome Project populations. Principal component analysis was performed using the -–pca argument of PLINK v2.0 [82,83]. We find that, despite being recruited in Chicago, Tibetans in our cohort showed strong genetic similarity to other Tibetan populations, in particular to those from the Western Plateau (S1 Fig).

#### Cell type characterization and differential expression from single Cell RNA-sequencing.

The cell ranger count pipeline of the 10X Genomics Cell Ranger 3.1.0 software package [84] was used to align reads to the transcriptome (GRCh38; Gencode 2019-09-05) and to generate matrices of UMI counts for each feature. The matrices from Cell Ranger were first processed using the workflow of the scRICA 0.0.0.9000 R package [85] to deconvolute doublets using the algorithm of DoubletDecon [86].The output of scRICA was then imported into Seurat 4.1.0 [33] where the data were further filtered using the parameters of the standard pre-processing workflow (cells were retained if 200 < gene count < 2,500 genes and if MT count < 5%) and then normalized. The R package Harmony 0.1.0 [87] was used to integrate samples and correct for data-specific conditions. Cell cycle scoring and regression was performed using the expression of G2/M and S phase markers following the Seurat workflow and using the list of cell cycle markers from Tirosh et al. 2015 [88].Seurat was then used to perform cell clustering using the Harmony embeddings. Cell cluster identification was based on endothelial cell canonical marker expression. All R packages for single cell analyses were run in R 4.1.0.

Differential gene expression analysis between KO and WT (5 independently isolated clones of each genotype) was performed on pseudo bulk data using the R package DESeq2 v1.34.0 [89] by first aggregating read counts of those clusters identified as EC in the single cell analysis for each sample. The final set of genes analyzed were those protein coding genes with at least 10 reads across all samples and those that passed the average expression threshold set by the independent Filtering function of DESeq2. *P*-values were corrected for multiple tests using the Benjamini-Hochberg method. All VST transformed counts from clusters 2/4 which can be found in S10 Table.

#### Differential expression (DE) analysis from bulk RNA-sequencing data.

Analysis was performed using a standard RNA-seq pipeline. Reads were aligned using STAR and read counts performed with RSEM using standard settings [90,91]. Gene filtering was performed with edgeR (v 3.32.1) filterByExpr [92]. In order to remove measured and unmeasured sources of variation from the data, sex-based effects were removed using Combat-Seq [93] while unknown variation was incorporated into the regression model by calculating surrogate variables using SVAseq [51]. iPSC differential expression analysis was performed using standard limma [94] and edgeR pipelines with a calculated 5SVs. For the paired differential expression analysis of iPSC-ECs in normoxia and hypoxia analysis was performed with dream (variancePartition v. 1.20.0 R package) following normalization using voomWithDreamWeights()[50]. The model: ~0 + (1|sample_ID) + population + condition + popuplation: condition + 10SV was used, separately setting normoxia and hypoxia as baseline to retrieve pop DE in both conditions. Sources of variation in the data were assessed by PCA, with individual lines found to contribute the most variation (PC1, 29%) followed by hypoxia treatment (PC2, 14%) (S13 Fig). All VST transformed counts can be found in supplemental S11 (iPSC) and S12 (iPSC-EC) Tables.

#### Cell-type deconvolution in population-wide panel of iPSC-ECs.

A custom signature matrix was built from the read count matrix of the approximately 9,700 cells of one replicate of the WT EC single-cell data using the “Create Signature Matrix” function of the online tool CIBERSORTx (https://cibersortx.stanford.edu/runcibersortx.php) Cell fractions were then independently imputed in the normoxia and hypoxia treated iPSC-derived EC samples using the generated signature matrix and the raw read count matrix from the bulk EC RNAseq data using the “Impute Cell Fractions” function of CIBERSORTx. We pooled cell fractions from the cluster 2 and 4 signatures to assess proportion of endothelial cells analogous to those assessed by scRNA-seq (S5 Table). We find no significant inter-population differences in cell-type composition in this cluster, nor any other cluster-imputed cell fraction (S5 Table and S8D Fig). To assess whether we needed to additionally account for cell-type composition in analysis we correlated fraction of cells in cluster 2/4 with surrogate variables included in the regression model. W1, the top surrogate variable in the model was highly correlated with cluster 2/4 proportion, effectively accounting for cell-type composition differences between lines (S8E Fig). We additionally assessed correlation of cell type fraction in all clusters assigned by CIBERSORTx with PluriTest scores to ascertain whether pluripotency potential of the starting cell line contributed to differentiated cell type proportion. We found no evidence of correlation between PluriTest score and any cluster-defined cell type (S8B, S8C and S14 Figs).

#### Enrichment analyses.

All enrichment analyses were performed using EnrichR [95–97] with a provided background universe. Briefly, EnrichR performs standard enrichment analysis and outputs a *p*-value using fisher’s exact test, and an adjusted *P*-value using Banjamini-Hochberg correction for multiple testing. Results were reported as significant if the adjusted *p*-value was < 0.05. Gene Ontology (GO) [98] and MSigDB [99] gene sets were included in analysis. For each independent analysis the background selected included all expressed genes in a given RNA-seq experiment.

### Polygenic adaptation analyses

We applied two different tests of polygenic adaptation. The first is a pairwise population test that follows [65] by considering the mean allele frequency difference between two populations for trait-increasing alleles of a set of SNPs associated with a trait. We used SNP frequencies estimated in a sample of 344 unrelated Tibetan individuals [6] and from 1 KG CHB to calculate the mean frequency difference between these populations for trait-increasing alleles. To test for significance of this statistic, we computed a null distribution of mean frequency differences for 10,000 sets of matching random SNPs, where a random SNP is considered to match a GWAS SNP if it falls in the same mean minor allele frequency bin of size 0.02. The empirical one-sided *p*-value was estimated as the proportion of random SNP sets with a mean allele difference greater than or equal to the observed one.

The second test is the outlier population method of [66]. This tests whether an estimated “genetic value,” calculated as a weighted sum of the population allele frequencies over the GWAS SNPs where the weights are the SNP effect sizes, for a group of populations deviates significantly from that expected under the null given other groups of populations. Here we test whether the regional group defined by Tibetan and Sherpa populations is an outlier given 25 1 KG populations defining 5 continental regions: Africa (YRI, LWK, GWD, MSL, ESN), Europe (CEU, GBR, IBS, TSI, FIN), South Asia (PJL, GIH, ITU, STU, BEB), East Asia (CDX, KHV, CHS, CHB, JPT), and the Americas (MXL, PUR, CLM, AYM, HUI). We used SNP frequencies estimated from 344 unrelated Tibetans as well as from 101 unrelated Sherpa from Jeong 2018 et al [6]. To calculate the genetic covariance matrix of populations and for generating 5,000 sets of matched random SNPs, we sampled random SNPs matching each of the GWAS SNPs by minor allele frequency bin of size 0.02 in the Tibetan population and by the B-value bin of size 100 (values ranging from 0 to 1,000) [100]. We sampled up to several thousands of random SNPs per GWAS SNP to obtain around 100,000 random SNPs in total.

The SNP-trait association data for both polygenic adaptation tests were taken from the autosomal GWAS results for 57 quantitative traits from the BioBank Japan Project [101]. We tested GWAS SNPs at a genome-wide significant *p*-value threshold of *p* < 1e-9 and a more liberal threshold of *p* < 1e-6. To obtain sets of independent markers, we selected one SNP per linkage disequilibrium block using the East Asian LD blocks identified by [102].

## Supporting information

S1 Fig Genetic Ancestry Analysis of TAC Tibetan samples. A) ADMIXTURE plot of the 10 TAC individuals used to generate the iPSC panel alongside publicly available data for 20 ethnically Tibetan individuals from two districts in Nepal (10 Mustang and 10 Gorkha district) [6] and 97 ethnically Tibetan individuals from 10 sampling sites across the Plateau (5 Gannan, 12 Chamdo, 4 Xunhua, 20 Gangcha, 9 Lhasa, 8 Nagqu, 10 Yajiang, 10 Shigatse, 10 Xinlong, and 9 Shannan) [28]. Additionally, 4 populations from the 1 KG dataset are included: Han Chinese in Beijing, China (CHB), Gujarati Indians in Houston, Texas, USA (GIH), Kinh in Ho Chi Minh City, Vietnam (KHV), and Japanese in Tokyo, Japan (JPT). K = 4 was selected as the number of admixture components which best distinguished the 1 KG populations while having the second lowest cross-validation error (see Methods). B) PCA plot showing the same populations depicted in A. In both plots (A and B), TAC individuals show clear affinities with other Tibetan individuals, particularly those from Nepal and the Western Plateau.
(TIF)

S2 Fig Functional and transcriptional QC of iPSC panel. A) Fluorescent staining shows spontaneous generation of endoderm, mesoderm, and ectoderm germ layers in embryoid body assay. These images were generated at 10x magnification on the EVOS FL Digital Inverted Microscope (Advanced Microscopy Group). Images for all 20 iPSC lines can be found in Appendix on 10.5281/zenodo.14552961). Normalized RNA-seq expression values of 6 pluripotency factors compared between populations. No significant differences between populations were found.
(TIF)

S3 Fig Immunofluorescent staining of two representative samples of iPSC-derived vascular endothelium in one Tibetan (TAC801) and one Han Chinese (CHB608) cell line. CD31 encodes an endothelial cell surface marker (also called PECAM1), VWF is a canonical endothelial protein known to cluster into Weibel-Palade bodies which can be seen as foci in the image. Nuclear DNA is marked with Hoechst DNA stain.
(TIF)

S4 Fig Differentiation of iPSCs into vascular endothelium. A) Diagram of iPSC to EC differentiation protocol (Created in BioRender. Di Rienzo, A. (2025) https://BioRender.com/x16t096) [30]. iPSCs were first induced to differentiate into mesoderm using a GSK3 inhibitor/WNT activator (CHIR99021) and BMP4. Then differentiated into endothelium using VEGF-A and forskolin. Following differentiation, cells were either harvested for scRNA-seq or separated using an anti-CD144/CDH5 bead pulldown for bulk RNA sequencing. B) UMAP plot depicts *CDH5* expression and cell-type clusters identified by Seurat. C) Violin plot shows that highest *CDH5* expression can be found in clusters 2 and 4, which were used to define the endothelial subset in all downstream analyses. D) Correlation of the *Z*-scores of DE genes in single cell clusters identified by Seurat. While clusters 2–5 all show significant correlation, 2 and 4 are the most highly correlated and were grouped in subsequent analyses. E) Correlation of *Z*-scores for different pooling schemes in DE analysis. We pseudobulked cells either from clusters 2–5 or from clusters 2 and 4 only and found the results were highly correlated with one another. For all subsequent analyses, the pool of clusters 2 and 4 was used.
(TIF)

S5 Fig Successful differentiation of iPSCs into Endothelial cells across population panel. A) Heatmap depicting unsupervised clustering of iPSC and iPSC-derived endothelium RNA-seq data in relation to a manually curated set of iPSC and Endothelial markers. iPSC markers include pluripotency genes POU5F1, LIN28A, NANOG, and SOX2, and highly expressed hiPSC and ESC genes CDH1 and ESRG. EC genes include endothelial markers *KDR, FLT1, CLDN5, HEY1, CD34, ECSCR, CDH5,* and *GJA5*. Unsupervised clustering performed using vst transformed counts, Euclidian distances, and average clustering method in Pheatmap [103]. B) boxplots of a subset of iPSC markers and C) EC markers showing vst counts between the two cell types.
(TIF)

S6 Fig Loss of ENH5 in iPSC-derived endothelial cells dysregulates HIF-2α target genes. A) Volcano plot shows 49 DE genes between ENH5 KO and WT endothelial cells. Analysis was performed using pseudobulk counts from cluster 2/4. Top DE genes are labeled. Blue indicates genes which are significantly down in KO compared to WT replicates (adjusted *p* < 0.05). Red indicates genes which are significantly up in KO compared to WT replicates (adjusted *p* < 0.05). *EPAS1*, which is not found to be significantly DE at transcriptome wide significance (*p* = *0.0033, adjusted p* = 0.25951961), is also labeled in purple. B and C) Plots depicting correlation between all logFC expression differences of all genes found to be differentially expressed in a HIF-1α or HIF-2α overexpression system in human primary endothelium (taken from Downes et al., 2018 [36]) and all gene expression differences between WT and ENH5 KO hiPSC-ECs. B) shows a significant negative correlation between the effect of HIF-2α overexpression in primary endothelium and ENH5 knockout in iPSC-derived endothelium while no such correlation is seen with C) The effect of HIF-1α overexpression, thus demonstrating the HIF-2α-mediated effects of ENH5 deletion.
(TIF)

S7 Fig Enrichment Analysis performed using enrichR [95–97] of all DE genes between ENH5 KO and WT (adjusted p or lfsr < 0.1) in iPSC-ECs, mouse lung, and mouse left atrium. Y axis shows the GSEA term, x axis shows the tissue in which DE was ascertained.
(TIF)

S8 Fig Accounting for sources of variation in cell-type composition of iPSC-ECs. A) There is a strong intra-line correlation in cell type composition between conditions. B) PluriTest scores show no correlation with proportion of cells identified as endothelial cells by CIBERSORTx in Normoxia or C) Hypoxia. D) We find no population-specific differences in the proportion of endothelial cells (defined by the cluster 2 and 4 transcriptional profile, two-sided Wilcoxon rank sum test, *p* > 0.05) between populations. E) We find a strong positive correlation between surrogate variable 1 (W1) inferred by SVAseq and % of endothelial cells (defined by cluster 2/4). This suggests W1 is capturing cell composition variation amongst samples in our analysis.
(TIF)

S9 Fig DE genes between populations in iPSC-ECs exposed to normoxia and hypoxia. A) *EPAS1* is significantly down-regulated between populations in hypoxia(adjusted p = 0.00102) though not in normoxia (adjusted p = 0.193). B) Correlation of logFC between populations of the 47 genes differentially expressed in both normoxia and hypoxia. C and D) A selection of two DE genes between populations in both conditions that belong to the enriched Inflammatory Response gene set (MSig DB, S6 Table).
(TIF)

S10 Fig Gene set enrichment analysis of DE genes in population-panel of iPSC-ECs. This dot plot depicts the GSEA results for all gene sets examined in the iPSC-EC panel including those DE between populations in hypoxia, normoxia, and both; as well as those involved in an enhanced or blunted response amongst the TAC lines. GSEA terms are shown on the y axis with the database in parentheses. The gene set used in analysis is shown on the x axis, corresponding to S7 Table.
(TIF)

S11 Fig Polygenic adaptation analyses plots for all traits with significant results. Titles of individual plots indicate both the trait and the GWAS cutoff of SNPs included in the test.
(TIF)

S12 Fig Comparison of all RNA-seq analysis performed with and without lines containing mosaic chromosomal abnormalities. In all cases, there was strongly significant positive correlation in z-scores. Therefore, the full set was included in the final analyses presented.
(TIF)

S13 Fig Known sources of variation in iPSC-derived ECs. A) Population-based differences are most correlated with PC3 which captures 2% of variation in the RNA-seq data. This relatively low percent is expected given the closely related populations and shared cell type. Meanwhile, the impact of hypoxia is captured in PC2, which accounts for 14% of variation. C) PC13 captures sex-based differences in gene expression (11% variance). Inter-line variation accounts for 29% of variance in expression. D) We find strong correlation in PC1 across conditions indicating sample-specific differences are driving this PC and remain consistent in normoxia and hypoxia.
(TIF)

S14 Fig No correlation between the proportions of cells assigned to each cluster in the bulk RNAseq data in iPSC-ECs and PluriTest Scores in iPSCs. Below are shown the correlation coefficients and p values for the correlation between the pluripotency score assigned by PluriTest to the original iPSC lines and the cell-type proportion of each cluster defined by CIBERSORTx in the iPSC-EC population panel. Results are shown separately in both normoxia and hypoxia for each cluster. None show significant correlations to pluripotency score.
(TIF)

S1 Table QC results and metadata for Tibetan and Han Chinese panel. Columns include sample ID, clone ID, population, sex, conversion batch (when cells were converted from feeder to feeder-free iPSCs), freeze-thaw survival, staining of endoderm, mesoderm, and ectoderm in embryoid body assay (for full imaging results see Appendix on 10.5281/zenodo.14552961), results from PluriTest, and karyotyping.
(XLSX)

S2 Table Differential expression results from performing RNA-seq in the iPSC panel comparing TAC – CHB. (XLSX)

S3 Table The number of cells in the two clusters identified as vascular endothelial cells in single cell RNA-sequencing. (XLSX)

S4 Table Differential expression results of ENH5 KO-WT iPSC ECs from cluster 2/4. (XLSX)

S5 Table Results of cell-type deconvolution of the iPSC-EC bulk RNAseq data using CIBERSORTx [52]. We used the expression profiles from each cluster identified in scRNA-seq of a WT replicate to deconvolute the cell type composition of all 20 iPSC-EC lines in hypoxia and normoxia. Each column represents the percentage of cells mapped to each cluster identified by single cell RNA-seq. Clusters 2 & 4 represent endothelial cells as defined by canonical marker gene expression. While proportions vary by line, we find no significant differences between populations in either condition.
(XLSX)

S6 Table Differential expression results for DREAM analysis of TAC and CHB iPSC-EC RNA-sequencing in normoxia and hypoxia. (XLSX)

S7 Table Gene set enrichment results for different groups of DE genes in the DREAM analysis of TAC and CHB iPSC-EC RNA-sequencing in normoxia and hypoxia. Analyses were performed with enrichR using the full set of expressed genes in the iPSC-EC data as a background.
(XLSX)

S8 Table Polygenic adaptation analysis results for the pairwise and multi-population tests for polygenic adaptation (see Methods). For each trait from BBJ the results of each test are reported at two GWAS significance cutoffs for inclusion of SNPs in the test. The SNP no. column shows how many SNPS are included in the test at this significance threshold. Significant *p*-values are highlighted in green (figures for all significant Multi-population tests in S6 Fig). In the pairwise test, a direction of effect can be inferred for the target population (i.e., does selection lead to an increase or decrease in the trait compared to the control population). This direction is indicated by the sign of the *p*-value. NA is reported when there are insufficient SNPs to carry out the test.
(XLSX)

S9 Table RNA sample and data quality metrics for all bulk RNA-sequencing. This table shows sample quality (RIN) as well as data quality metrics extracted from RSEM for all bulk RNA-sequencing. The top 20 samples are iPSCs, and the remainder are iPSC-ECs. N indicates normoxia, H indicates Hypoxia.
(XLSX)

S10 Table VST transformed counts data for pseudobulked clusters 2 and 4 in iPSC-EC scRNAseq data. Each row is a replicate in which KO indicated ENH5 knockout and WT indicates a vehicle treated wildtype line. All cells come from individual CHB633.
(XLSX)

S11 Table VST transformed counts of panel-wide iPSC transcriptomic data. Column names indicate sample ID, row names indicate ensemble gene ID.
(XLSX)

S12 Table VST transformed counts of panel-wide iPSC-EC transcriptomic data. Column names indicate sample ID; N and H indicate normoxia or hypoxia respectively; row names indicate ensemble gene ID.
(XLSX)
