# Supplementary figures and images for: Transcriptomic analysis of iPSC-derived endothelium reveals adaptations to high altitude hypoxia in energy metabolism and inflammation
Source: PLoS Genet. 2025 Feb 10;21(2):e1011570. doi: 10.1371/journal.pgen.1011570 (PMC11809796; doi:10.1371/journal.pgen.1011570)

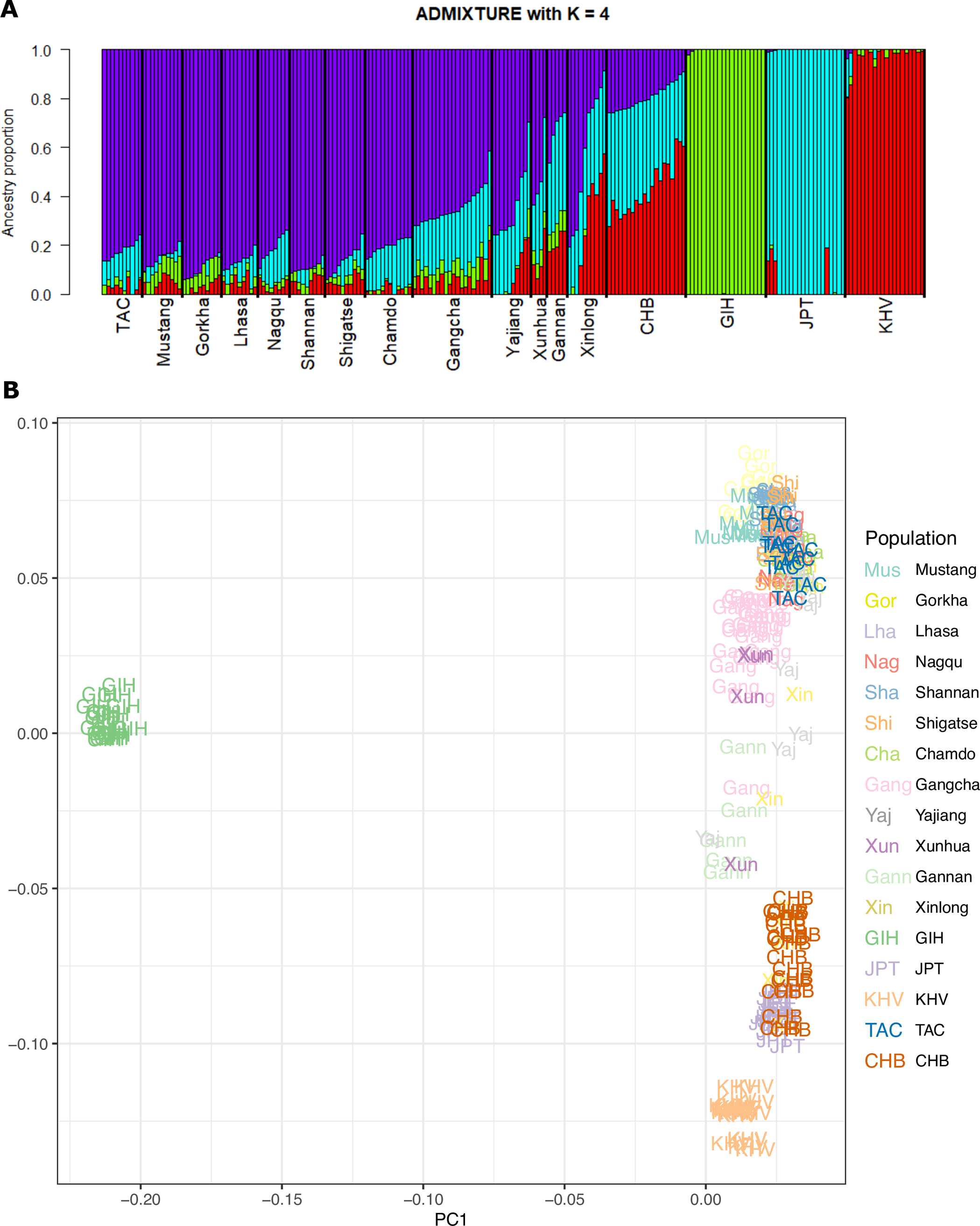

Supplement: S1 Fig — A) ADMIXTURE plot of the 10 TAC individuals used to generate the iPSC panel alongside publicly available data for 20 ethnically Tibetan individuals from two districts in Nepal (10 Mustang and 10 Gorkha district) [6] and 97 ethnically Tibetan individuals from 10 sampling sites across the Plateau (5 Gannan, 12 Chamdo, 4 Xunhua, 20 Gangcha, 9 Lhasa, 8 Nagqu, 10 Yajiang, 10 Shigatse, 10 Xinlong, and 9 Shannan) [28]. Additionally, 4 populations from the 1 KG dataset are included: Han Chinese in Beijing, China (CHB), Gujarati Indians in Houston, Texas, USA (GIH), Kinh in Ho Chi Minh City, Vietnam (KHV), and Japanese in Tokyo, Japan (JPT). K = 4 was selected as the number of admixture components which best distinguished the 1 KG populations while having the second lowest cross-validation error (see Methods). B) PCA plot showing the same populations depicted in A. In both plots (A and B), TAC individuals show clear affinities with other Tibetan individuals, particularly those from Nepal and the Western Plateau. (TIF) [file pgen.1011570.s001.tif]

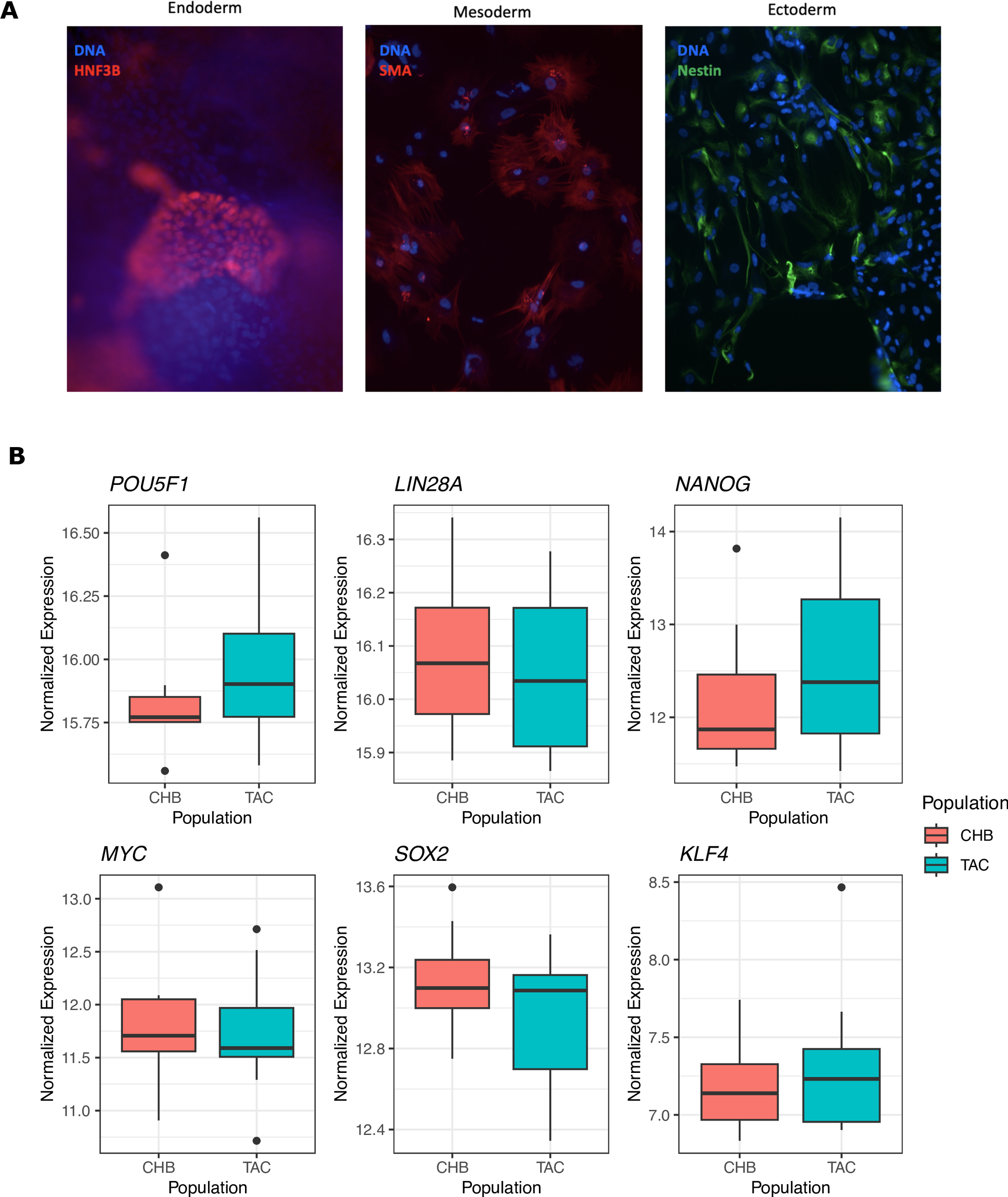

Supplement: S2 Fig — A) Fluorescent staining shows spontaneous generation of endoderm, mesoderm, and ectoderm germ layers in embryoid body assay. These images were generated at 10x magnification on the EVOS FL Digital Inverted Microscope (Advanced Microscopy Group). Images for all 20 iPSC lines can be found in Appendix on 10.5281/zenodo.14552961). Normalized RNA-seq expression values of 6 pluripotency factors compared between populations. No significant differences between populations were found. (TIF) [file pgen.1011570.s002.tif]

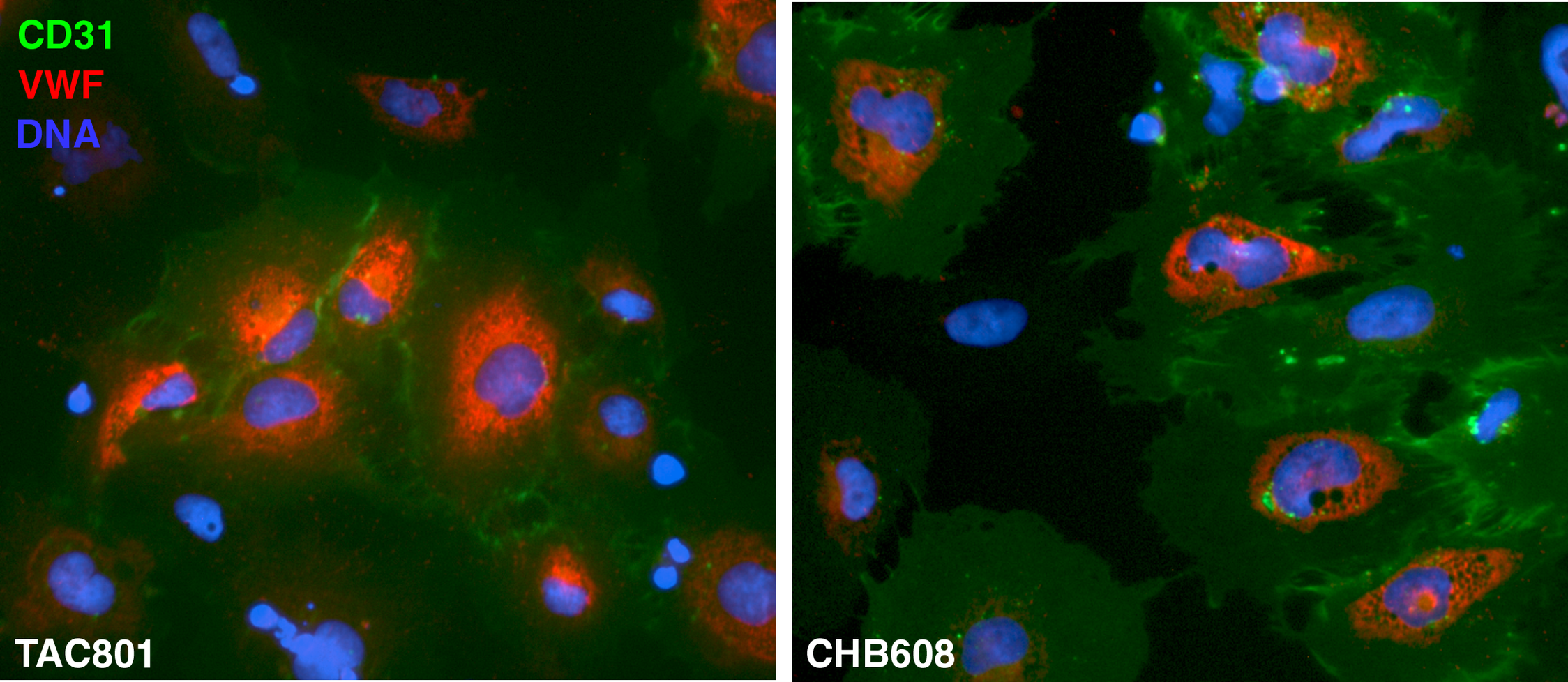

Supplement: S3 Fig — CD31 encodes an endothelial cell surface marker (also called PECAM1), VWF is a canonical endothelial protein known to cluster into Weibel-Palade bodies which can be seen as foci in the image. Nuclear DNA is marked with Hoechst DNA stain. (TIF) [file pgen.1011570.s003.tif]

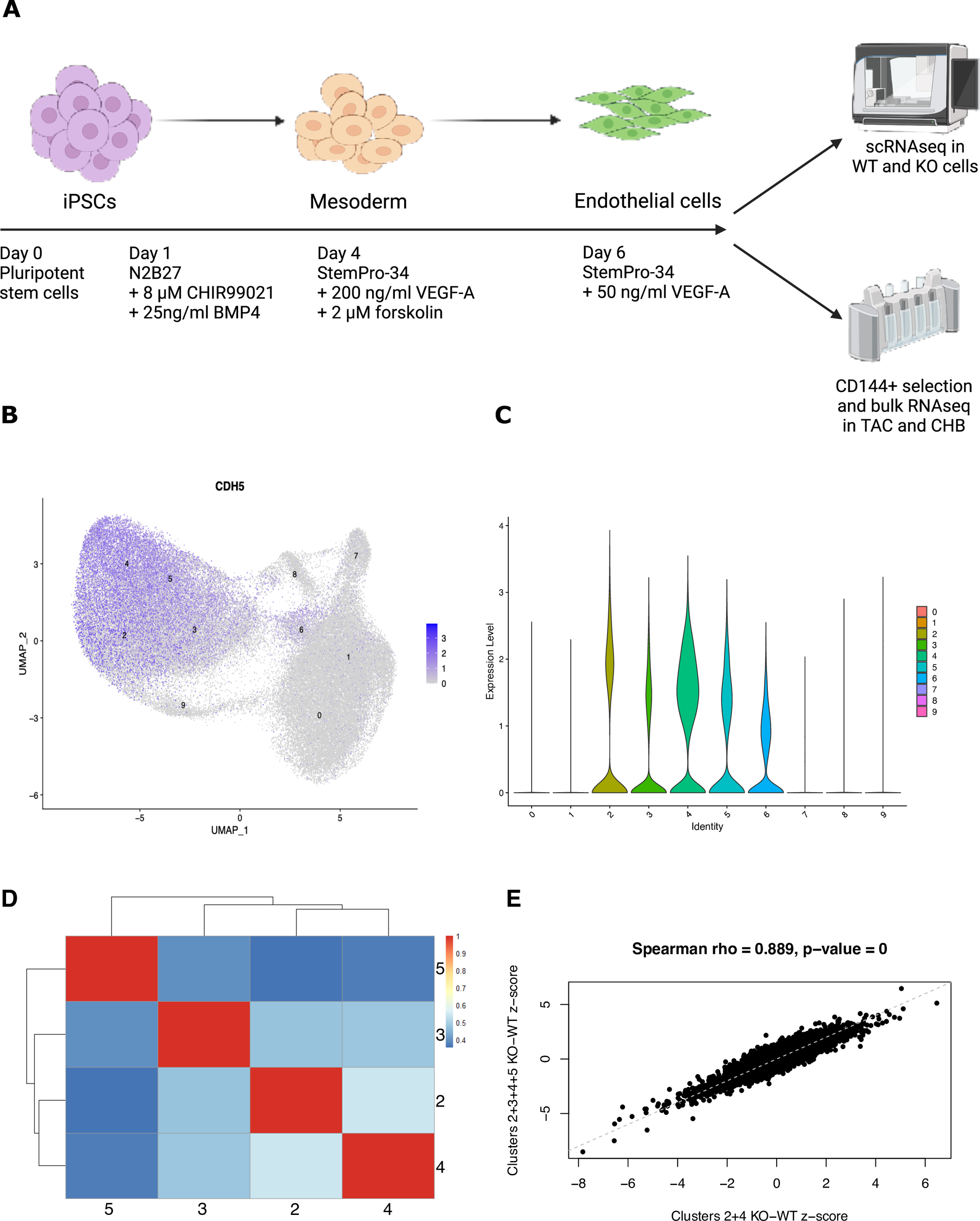

Supplement: S4 Fig — A) Diagram of iPSC to EC differentiation protocol (Created in BioRender. Di Rienzo, A. (2025) https://BioRender.com/x16t096) [30]. iPSCs were first induced to differentiate into mesoderm using a GSK3 inhibitor/WNT activator (CHIR99021) and BMP4. Then differentiated into endothelium using VEGF-A and forskolin. Following differentiation, cells were either harvested for scRNA-seq or separated using an anti-CD144/CDH5 bead pulldown for bulk RNA sequencing. B) UMAP plot depicts CDH5 expression and cell-type clusters identified by Seurat. C) Violin plot shows that highest CDH5 expression can be found in clusters 2 and 4, which were used to define the endothelial subset in all downstream analyses. D) Correlation of the Z-scores of DE genes in single cell clusters identified by Seurat. While clusters 2–5 all show significant correlation, 2 and 4 are the most highly correlated and were grouped in subsequent analyses. E) Correlation of Z-scores for different pooling schemes in DE analysis. We pseudobulked cells either from clusters 2–5 or from clusters 2 and 4 only and found the results were highly correlated with one another. For all subsequent analyses, the pool of clusters 2 and 4 was used. (TIF) [file pgen.1011570.s004.tif]

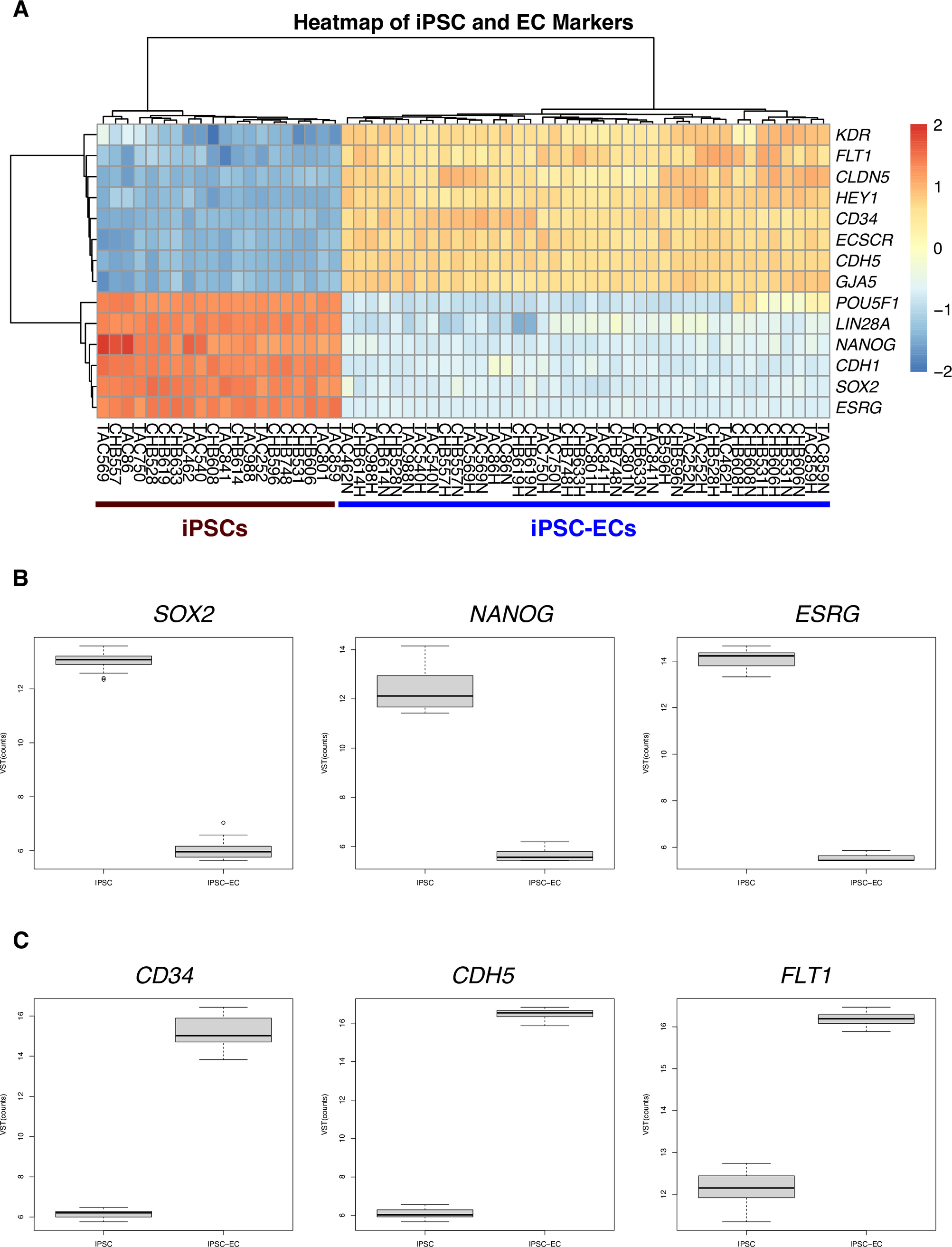

Supplement: S5 Fig — A) Heatmap depicting unsupervised clustering of iPSC and iPSC-derived endothelium RNA-seq data in relation to a manually curated set of iPSC and Endothelial markers. iPSC markers include pluripotency genes POU5F1, LIN28A, NANOG, and SOX2, and highly expressed hiPSC and ESC genes CDH1 and ESRG. EC genes include endothelial markers KDR, FLT1, CLDN5, HEY1, CD34, ECSCR, CDH5, and GJA5. Unsupervised clustering performed using vst transformed counts, Euclidian distances, and average clustering method in Pheatmap [103]. B) boxplots of a subset of iPSC markers and C) EC markers showing vst counts between the two cell types. (TIF) [file pgen.1011570.s005.tif]

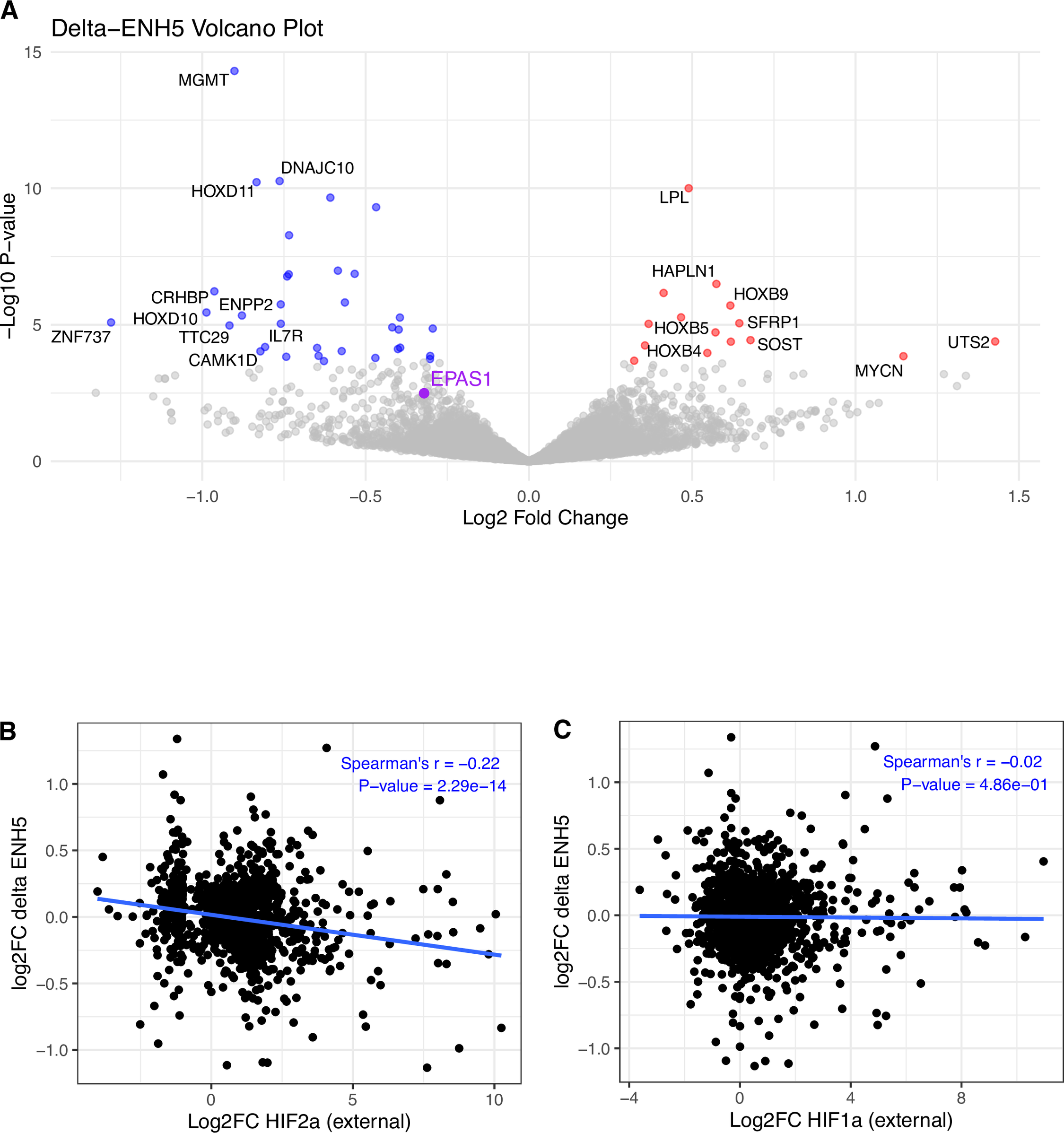

Supplement: S6 Fig — A) Volcano plot shows 49 DE genes between ENH5 KO and WT endothelial cells. Analysis was performed using pseudobulk counts from cluster 2/4. Top DE genes are labeled. Blue indicates genes which are significantly down in KO compared to WT replicates (adjusted p < 0.05). Red indicates genes which are significantly up in KO compared to WT replicates (adjusted p < 0.05). EPAS1, which is not found to be significantly DE at transcriptome wide significance (p = 0.0033, adjusted p = 0.25951961), is also labeled in purple. B and C) Plots depicting correlation between all logFC expression differences of all genes found to be differentially expressed in a HIF-1α or HIF-2α overexpression system in human primary endothelium (taken from Downes et al., 2018 [36]) and all gene expression differences between WT and ENH5 KO hiPSC-ECs. B) shows a significant negative correlation between the effect of HIF-2α overexpression in primary endothelium and ENH5 knockout in iPSC-derived endothelium while no such correlation is seen with C) The effect of HIF-1α overexpression, thus demonstrating the HIF-2α-mediated effects of ENH5 deletion. (TIF) [file pgen.1011570.s006.tif]

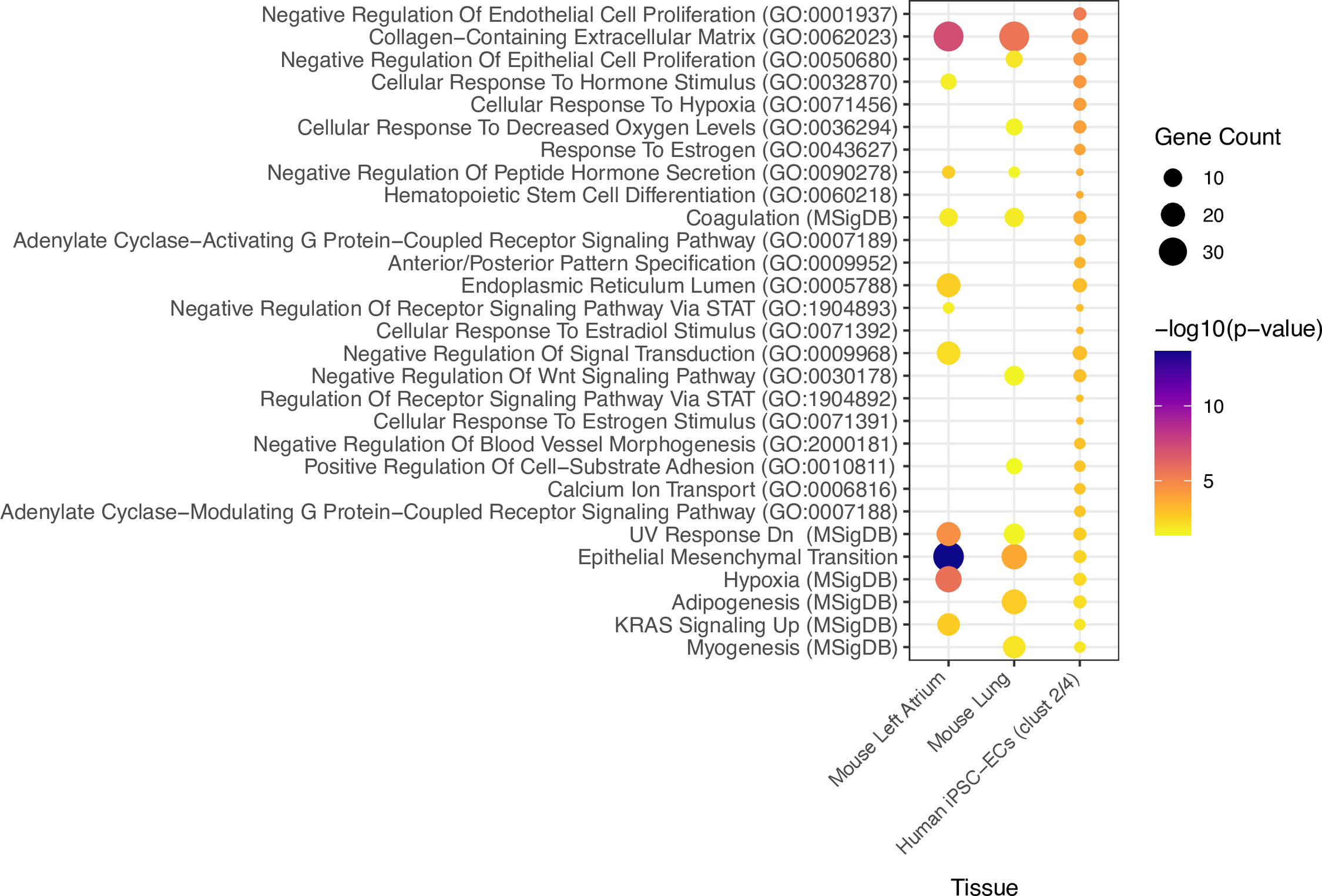

Supplement: S7 Fig — Y axis shows the GSEA term, x axis shows the tissue in which DE was ascertained. (TIF) [file pgen.1011570.s007.tif]

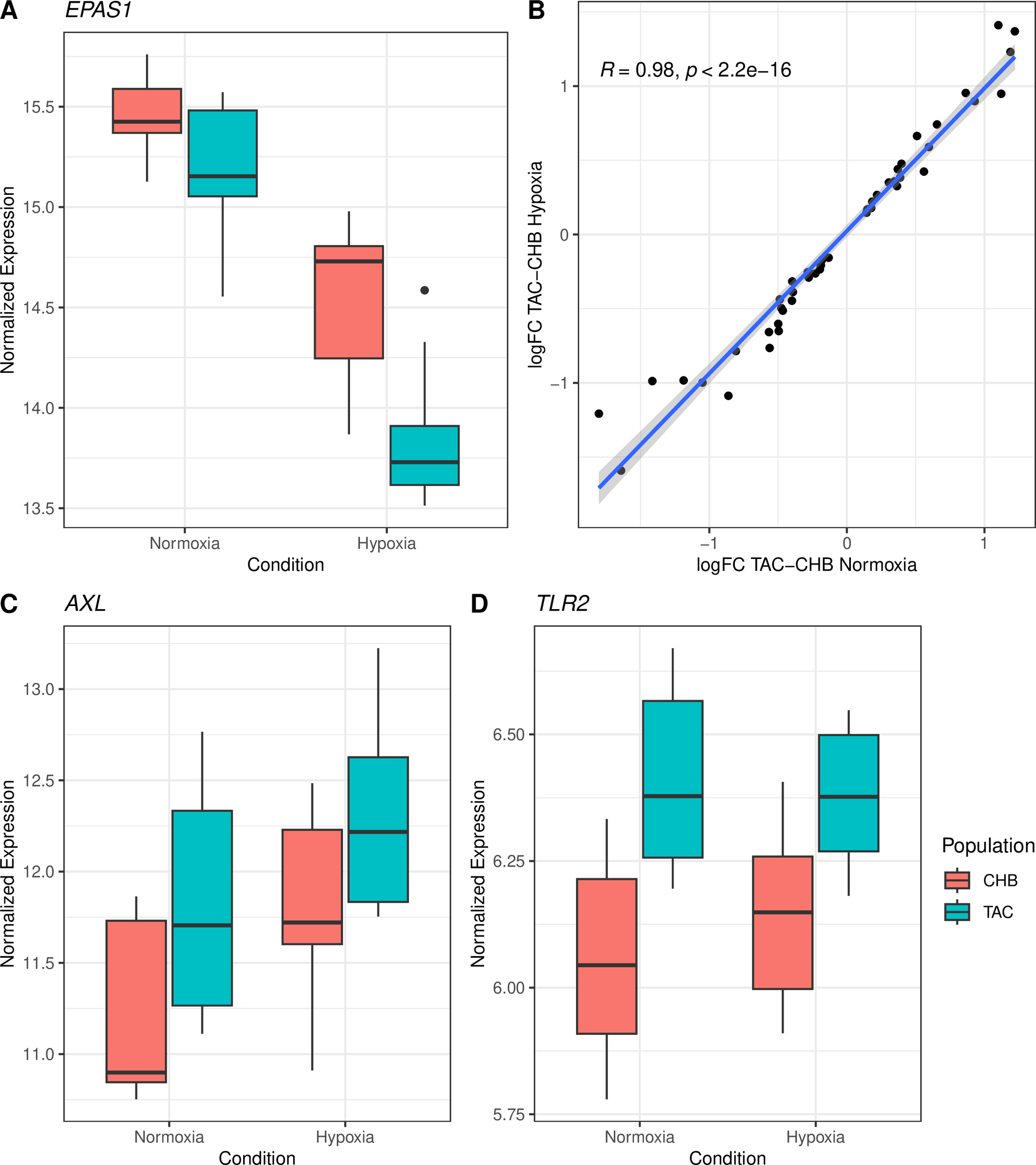

Supplement: S8 Fig — A) There is a strong intra-line correlation in cell type composition between conditions. B) PluriTest scores show no correlation with proportion of cells identified as endothelial cells by CIBERSORTx in Normoxia or C) Hypoxia. D) We find no population-specific differences in the proportion of endothelial cells (defined by the cluster 2 and 4 transcriptional profile, two-sided Wilcoxon rank sum test, p > 0.05) between populations. E) We find a strong positive correlation between surrogate variable 1 (W1) inferred by SVAseq and % of endothelial cells (defined by cluster 2/4). This suggests W1 is capturing cell composition variation amongst samples in our analysis. (TIF) [file pgen.1011570.s008.tif]

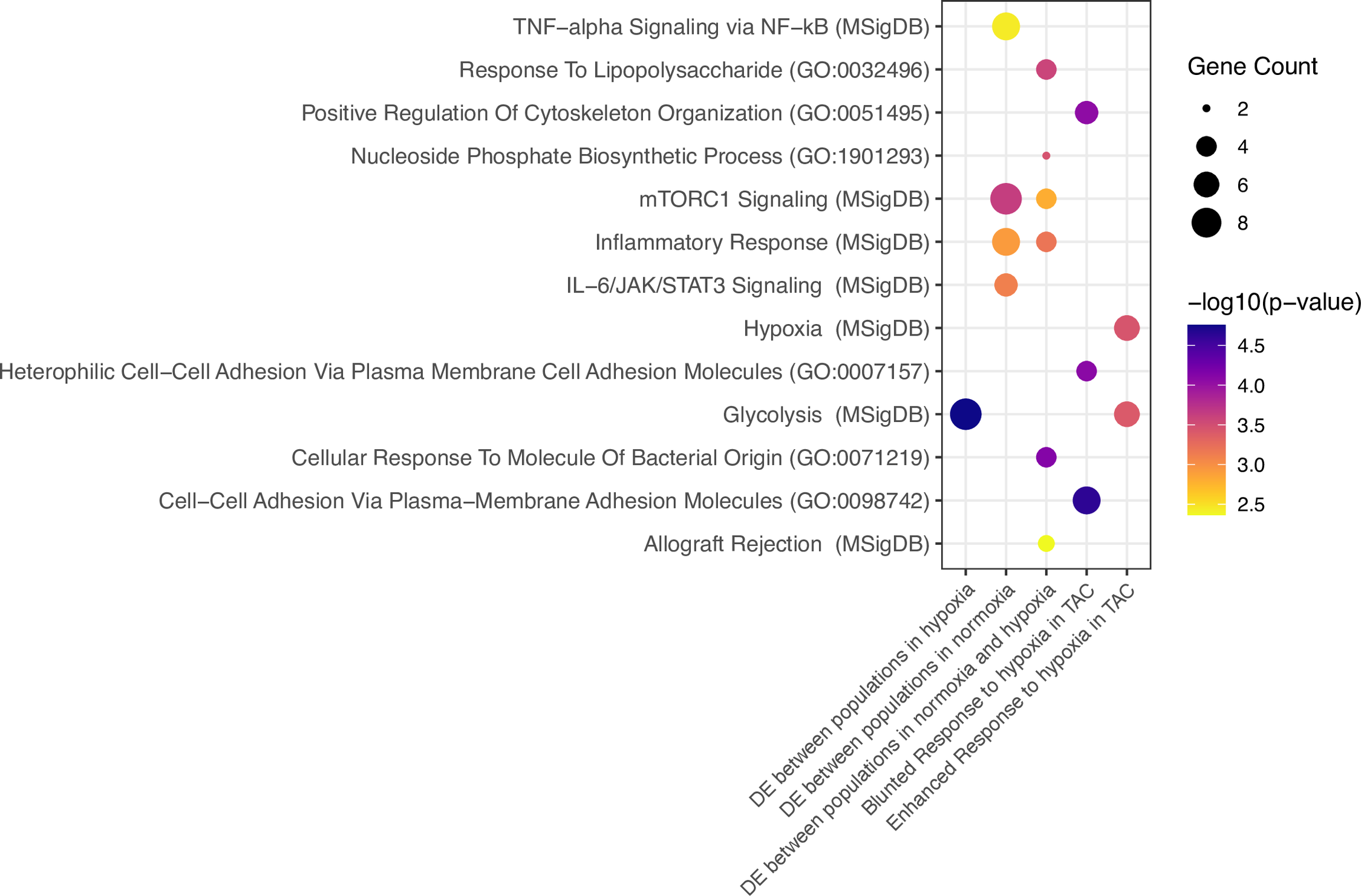

Supplement: S10 Fig — This dot plot depicts the GSEA results for all gene sets examined in the iPSC-EC panel including those DE between populations in hypoxia, normoxia, and both; as well as those involved in an enhanced or blunted response amongst the TAC lines. GSEA terms are shown on the y axis with the database in parentheses. The gene set used in analysis is shown on the x axis, corresponding to S7 Table. (TIF) [file pgen.1011570.s010.tif]

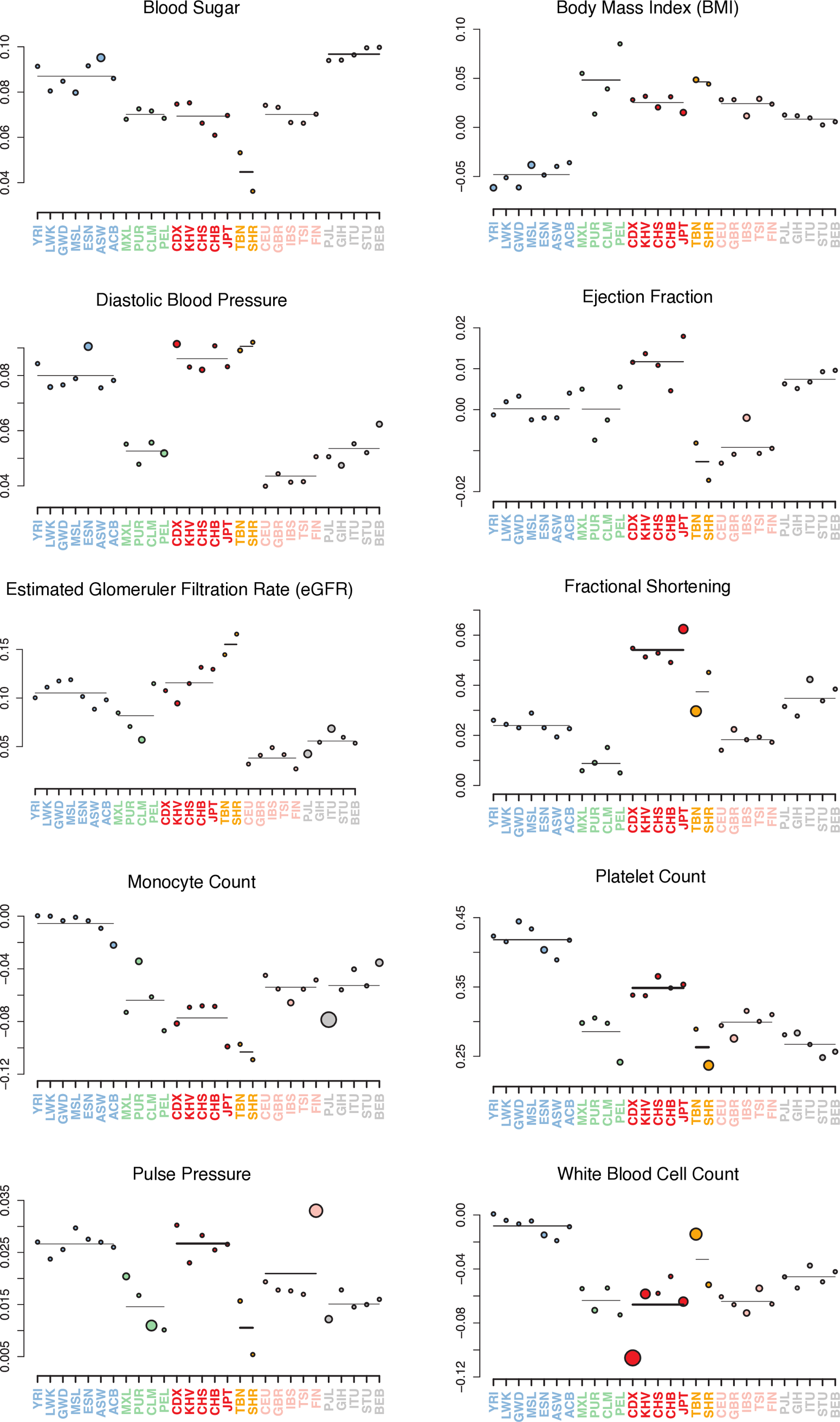

Supplement: S11 Fig — Titles of individual plots indicate both the trait and the GWAS cutoff of SNPs included in the test. (TIF) [file pgen.1011570.s011.tif]

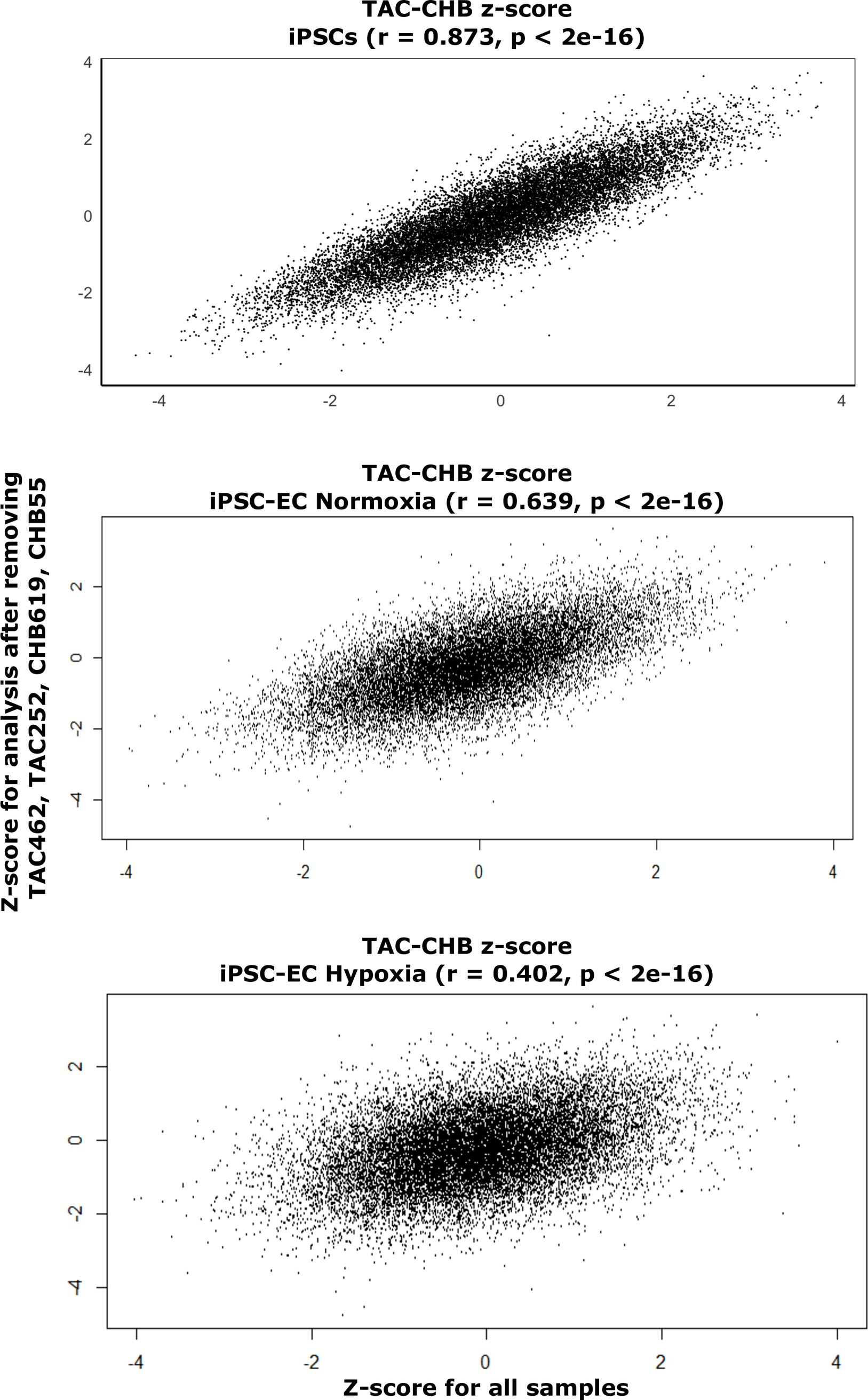

Supplement: S12 Fig — In all cases, there was strongly significant positive correlation in z-scores. Therefore, the full set was included in the final analyses presented. (TIF) [file pgen.1011570.s012.tif]

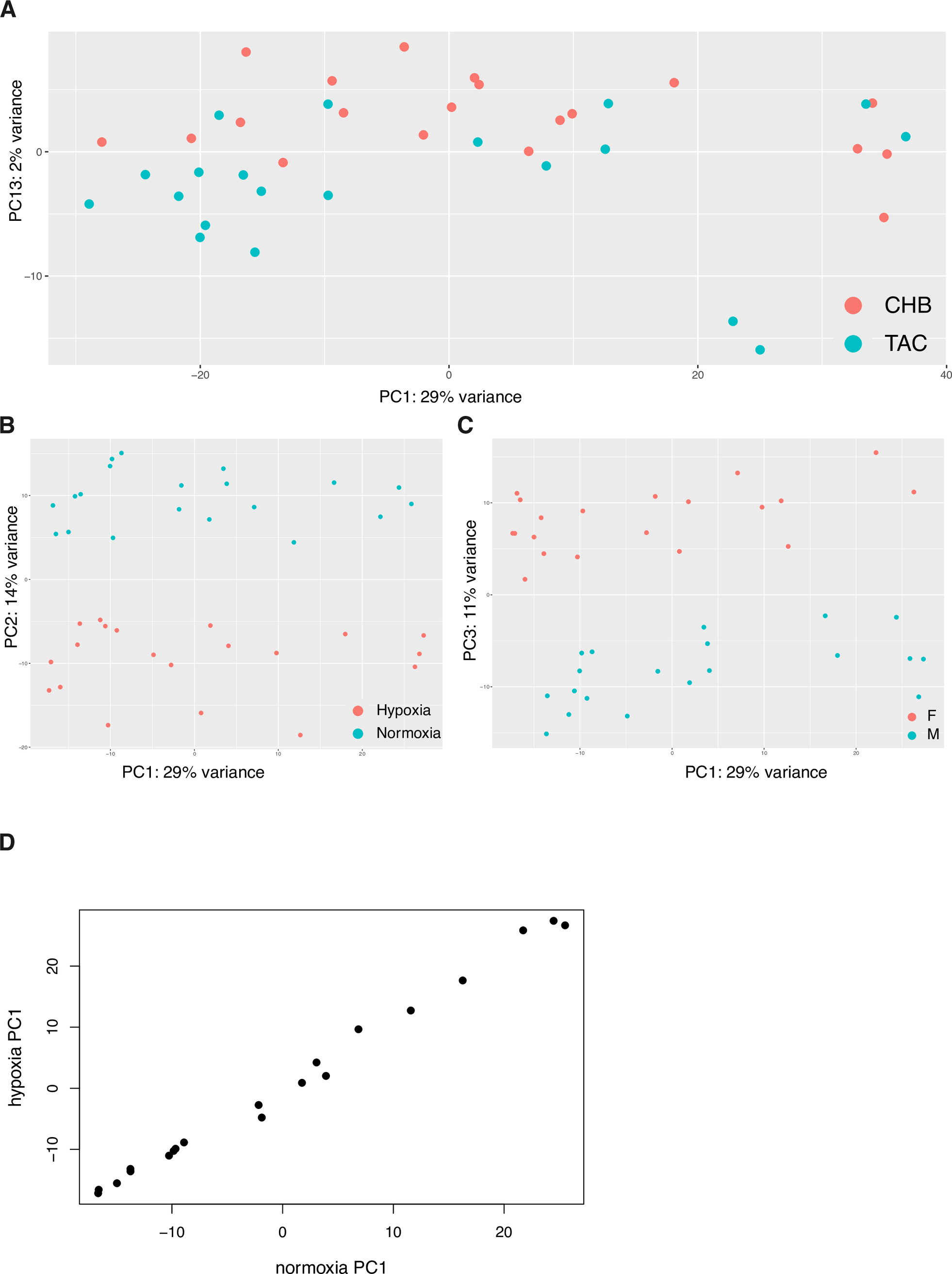

Supplement: S13 Fig — A) Population-based differences are most correlated with PC3 which captures 2% of variation in the RNA-seq data. This relatively low percent is expected given the closely related populations and shared cell type. Meanwhile, the impact of hypoxia is captured in PC2, which accounts for 14% of variation. C) PC13 captures sex-based differences in gene expression (11% variance). Inter-line variation accounts for 29% of variance in expression. D) We find strong correlation in PC1 across conditions indicating sample-specific differences are driving this PC and remain consistent in normoxia and hypoxia. (TIF) [file pgen.1011570.s013.tif]

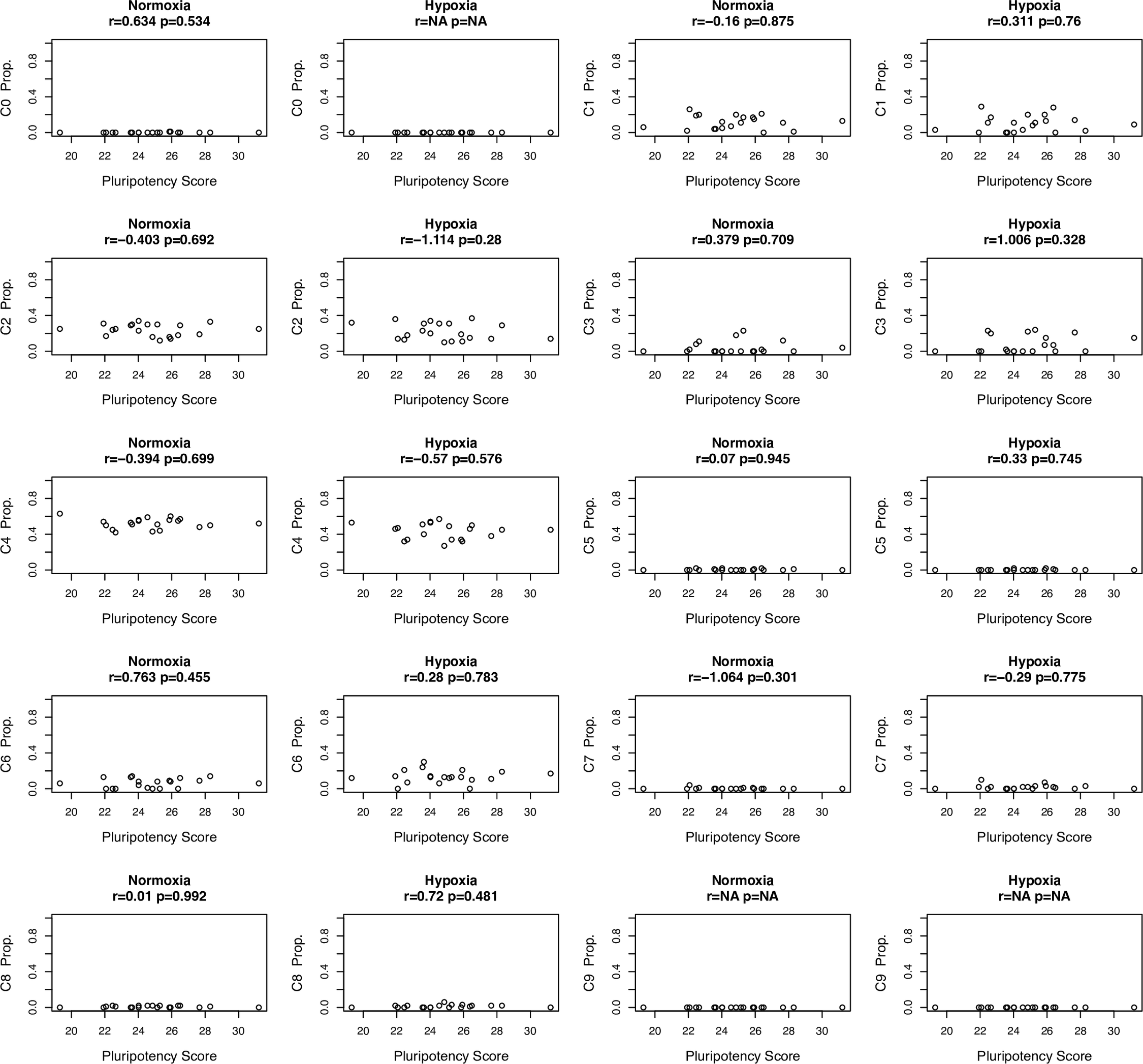

Supplement: S14 Fig — Below are shown the correlation coefficients and p values for the correlation between the pluripotency score assigned by PluriTest to the original iPSC lines and the cell-type proportion of each cluster defined by CIBERSORTx in the iPSC-EC population panel. Results are shown separately in both normoxia and hypoxia for each cluster. None show significant correlations to pluripotency score. (TIF) [file pgen.1011570.s014.tif]
